# Resveratrol-Mediated Regulation of Mitochondria Biogenesis-associated Pathways in Neurodegenerative Diseases: Molecular Insights and Potential Therapeutic Applications

**DOI:** 10.2174/1570159X20666221012122855

**Published:** 2023-04-12

**Authors:** Abdullah Shaito, Maryam Al-Mansoob, Salma M.S. Ahmad, Mohammad Z. Haider, Ali H. Eid, Anna Maria Posadino, Gianfranco Pintus, Roberta Giordo

**Affiliations:** 1 Biomedical Research Center, College of Medicine, Qatar University, Doha, 2713, Qatar;; 2 Department of Biomedical Sciences, College of Health Sciences, Qatar University, Doha, 2713, Qatar;; 3 Department of Biological and Environmental Sciences, College of Arts and Sciences, Qatar University, Doha, 2713, Qatar;; 4 College of Medicine, QU Health, Qatar University, Doha, 2713, Qatar;; 5 Department of Basic Medical Sciences, College of Medicine, QU Health, Qatar University, Doha, 2713, Qatar;; 6 Department of Biomedical Sciences, University of Sassari, 07100, Sassari, Italy;; 7 Department of Medical Laboratory Sciences, College of Health Sciences and Sharjah Institute for Medical Research, University of Sharjah, University City Rd, Sharjah, 27272, United Arab Emirates;; 8 College of Medicine, Mohammed Bin Rashid University of Medicine and Health Sciences, Dubai, 505055, United Arab Emirates

**Keywords:** Resveratrol, neurodegenerative diseases, mitochondria, mitochondrial biogenesis, polyphenols, central nervous system

## Abstract

Neurodegenerative disorders include different neurological conditions that affect nerve cells, causing the progressive loss of their functions and ultimately leading to loss of mobility, coordination, and mental functioning. The molecular mechanisms underpinning neurodegenerative disease pathogenesis are still unclear. Nonetheless, there is experimental evidence to demonstrate that the perturbation of mitochondrial function and dynamics play an essential role. In this context, mitochondrial biogenesis, the growth, and division of preexisting mitochondria, by controlling mitochondria number, plays a vital role in maintaining proper mitochondrial mass and function, thus ensuring efficient synaptic activity and brain function. Mitochondrial biogenesis is tightly associated with the control of cell division and variations in energy demand in response to extracellular stimuli; therefore, it may represent a promising therapeutic target for developing new curative approaches to prevent or counteract neurodegenerative disorders. Accordingly, several inducers of mitochondrial biogenesis have been proposed as pharmacological targets for treating diverse central nervous system conditions. The naturally occurring polyphenol resveratrol has been shown to promote mitochondrial biogenesis in various tissues, including the nervous tissue, and an ever-growing number of studies highlight its neuro-therapeutic potential. Besides preventing cognitive impairment and neurodegeneration through its antioxidant and anti-inflammatory properties, resveratrol has been shown to be able to enhance mitochondria biogenesis by acting on its main effectors, including PGC-1α, SIRT1, AMPK, ERRs, TERT, TFAM, NRF-1 and NRF-2. This review aims to present and discuss the current findings concerning the impact of resveratrol on the machinery and main effectors modulating mitochondrial biogenesis in the context of neurodegenerative diseases.

## 1 INTRODUCTION

Neurodegenerative disorders encompass a wide range of conditions caused by the progressive damage and loss of nerve cell functions and connections, ultimately affecting mobility, coordination, and mental functioning [1]. Symptoms vary depending on the affected brain regions; some neurodegenerative disorders mainly cause mobility defects, while others compromise memory and cognitive abilities [1, 2]. Neurodegenerative diseases affect millions of people worldwide and Parkinson's disease (PD), Alzheimer's disease (AD), Huntington’s disease (HD), and amyotrophic lateral sclerosis (ALS) are four of the most common neurodegenerative diseases [3]. Aging is the most significant risk factor, but systemic conditions such as inflammation and oxidative stress, as well as mitochondrial dysfunctions (which, along with oxidative stress, contribute to aging), are known to aggravate neurodegeneration [4-7]. Indeed, neurodegeneration’s crucial feature is cell death, where mitochondria, mainly through the intrinsic mitochondrial apoptotic pathway, act as important cell death regulators [8]. *Via* the mitochondrial respiratory chain activity, mitochondria are also significant contributors to reactive oxygen species (ROS) generation, which at physiological concentrations are crucial regulators of essential cell functions [7]. Moreover, functional mitochondria are important for activating proper stress reactions and maintaining metabolic homeostasis, which is correlated with lifespan extension and aging [9]. Therefore, although the mechanisms of pathogenesis of neurodegenerative diseases are still unclear, several lines of evidence show that perturbation of mitochondrial function and dynamics has an essential role. Because of their high energy demand, neurons are prone to injury and death resulting from dysfunctional mitochondria [9]. As a result, neurons possess several cellular programs designated to maintain mitochondrial quality and integrity by eliminating and substituting dysfunctional mitochondria with new functional ones; for instance, compromised mitochondria are subjected to autophagic degradation (mitophagy) and recent studies revealed that defects in mitophagy are associated with neurodegenerative diseases [10]. Besides, because of their dynamic nature, mitochondria undergo continual fusion and fission events which serve to maintain mitochondrial integrity and quantity, and are also important for ATP production, Ca^2+^ homeostasis, ROS production and regulation of apoptosis regulations [11, 12]. Indeed, mitochondrial fusion, the process where two separate mitochondria can fuse to form a larger one, prevents the permanent loss of essential mitochondrial components by retaining the contents of partially damaged mitochondria [11]. On the other hand, mitochondrial fission, the segregation into two new organelles, creates new mitochondria [13]. Both processes are balanced in the cell, and mitochondria use fission and fusion processes in response to signals like changes in energy and stress status [14]. Mitochondrial biogenesis, the growth and division of preexisting mitochondria, a biological process different from mitochondrial fusion, cycles and mitophagy, plays a determinant action in maintaining mitochondrial health and energetic homeostasis within the cell [9, 15]. Through this mechanism, cells can control mitochondria number [9, 15], indeed, the balance between the biogenesis rate and the removal of dysfunctional and old mitochondria is essential for maintaining an adequate and functional mitochondrial mass, as well as for ensuring an efficient brain synaptic activity [15]. Although all these processes work distinctly, they are interdependent and constantly controlled by shared effectors and regulators. Changes in this dynamism result in mitochondrial dysfunction and consequent defects in bioenergetics [12]. In this regard, since mitochondrial biogenesis is not only performed in association with cell division but can also be induced in response to increased energy demand, upon oxidative stress or to attenuate mitochondrial dysfunction, for example, it represents a promising therapeutic target for the treatment of neurodegenerative diseases [15, 16]. Moreover, a considerable number of regulatory proteins, transcription factors, and secondary mechanisms involved in mitochondrial biogenesis are good candidates for developing new therapeutics in the context of neurodegenerative diseases [17]. Accordingly, several mitochondrial biogenesis inducers have been approved by the U.S. Food and Drug Administration (FDA) as pharmacological agents for the treatment of various pathologies affecting the central nervous system [17-19]. Due to its neuroprotective effects and its ability to promote mitochondrial biogenesis in various tissues, most importantly the nervous tissue, an increasing number of studies highlighted the neuro-therapeutic potential of the naturally occurring polyphenol resveratrol [18, 20]. In this context, resveratrol showed to be able to prevent cognitive impairment and neurodegeneration in age-related diseases by reducing inflammation and ROS levels *in vitro* and in animal models, inducing autophagy of defective mitochondria *in vitro* and in animal models, and reducing neuronal cell death improving synaptic plasticity in animal models, explaining resveratrol behavioral benefits observed in animal models and clinical trials [21]. Besides, several *in vitro* and *in vivo* animal studies reported that resveratrol can increase mitochondria biogenesis by acting on its main effectors such as peroxisome-proliferator-activated γ co-activator-1α (PGC-1α), Sirtuin 1 (SIRT1), adenosine monophosphate protein kinase (AMPK), estrogen‐related receptor‐α (ERR‐α), telomerase reverse transcriptase (TERT), transcription factor A mitochondrial (TFAM), nuclear respiration factors 1 and 2 (NRF-1, NRF-2 [17, 18, 20]. This review aims to present the current state of knowledge on the mechanisms by which resveratrol modulates mitochondrial biogenesis in the context of neurodegenerative diseases. Moreover, the future perspectives for the employment of resveratrol as a therapeutic approach in the neurodegenerative diseases field will be discussed.

## 2 MITOCHONDRIA BIOGENESIS IN NEURONS AND MAIN EFFECTORS

**Fig. (1):**
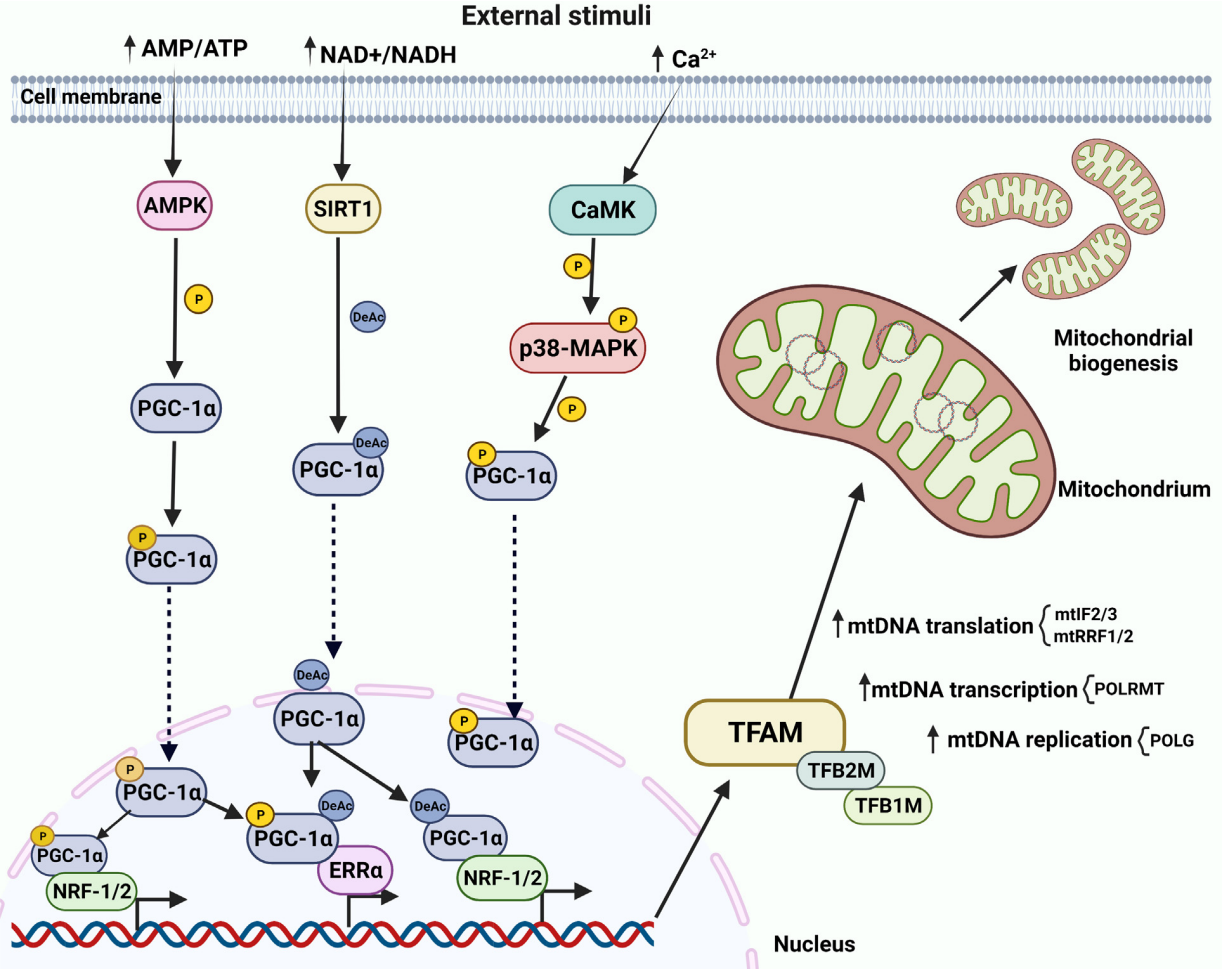
Overview of mitochondrial biogenesis in neurons. PGC-1α/NRF-1/2/TFAM is the main regulatory pathway of mitochondrial biogenesis. Activated PGC-1α stimulates nuclear transcription factors NRF-1, NRF-2, ERR‐α and the final effector TFAM, which (along with TFB1M, and TFB2M) controls mtDNA transcription and replication. Increased AMP/ATP ratio triggers the PGC-1α/NRF-1/2/TFAM pathway *via* AMPK activation, which in turn phosphorylates PGC-1α. Elevated Ca^2+^ levels induce mitochondrial biogenesis *via* CaMK activation, CaMK phosphorylates p38-MAPK, activating PGC-1α. Increased NAD+/NADH ratio induces mitochondrial biogenesis *via* SIRT1 activation, deacetylating PGC-1α. Dashed lines are for translocation.

Neurons are complex and highly polarized cells consisting of three main compartments, cell body, axon, and dendrites, that are further composed of sub‐compartments with essential roles for proper neuronal function. Each compartment requires a pool of mitochondria maintained by a constant mitochondrial turnover [22]. Mitochondrial biogenesis occurs mainly in the cell body, then mitochondria travel forth to the synapse to exert their functions, and when damaged, they travel back to the cell body for degradation. In some cases, such as for long cells, mitochondrial biogenesis can also occur at the periphery since this back and forth travel is not energetically favorable [23]. Being prokaryotic in origin, mitochondria have their own mitochondrial DNA and they can replicate independently of the host cell. Mitochondrial DNA (mtDNA) is composed of a double-stranded circular DNA molecule of approximately 16.5 kb, containing 37 genes encoding for the 13 subunits of the electron transport chain complexes I, III, IV and V. The remaining (over 1000) mitochondrial resident proteins are instead encoded by nuclear DNA (nDNA) [24-26]. Accordingly, mitochondrial biogenesis is a complex interplay of cellular and molecular processes that includes transcription and translation of mtDNA, and the synthesis, import and assembly of mitochondrial proteins encoded by the nDNA. All these processes require constant and precise communication between the nucleus and mitochondria, which ultimately leads to mitochondria replication [27]. Since neurons are high energy-demanding cells, they require tight regulation of Ca^2+^ concentration, which is essential for controlling oxidative phosphorylation and thereby contributing to the maintenance of cellular energy homeostasis [28]. Ca^2+^ concentration and AMP/ATP ratio are thus the main mitochondrial biogenesis triggers, while PGC-1α/NRF-1/2-TFAM is the main regulatory pathway (Fig. **1**) [23]. This pathway is initiated by the activation (*via* either phosphorylation or deacetylation) of PGC-1α, the master regulator of mitochondrial biogenesis. This, in turn, stimulates a series of nuclear transcription factors, including NRF-1 and NRF-2, ERR‐α, and the final effector TFAM [23, 27, 29], which, along with transcription factor B1 mitochondrial (TFB1M), and TFB2M, control mtDNA transcription and replication [30-33]. NRF-1 and NRF-2 also regulate other mitochondrial genes, such as those encoding proteins of the respiratory complex, cytochrome C oxidase subunit IV (COXIV), cytochrome c, and the heme biosynthetic pathway (globin) [30-33]. Mitochondrial replication is carried out by the mtDNA polymerase γ (POLG) which consist of a catalytic subunit encoded by the POLG gene and an auxiliary dimeric subunit encoded by the POLG2 gene (Fig. **1**) [34]. mtDNA transcription is instead catalyzed by the mitochondrial RNA polymerase POLRMT [35, 36], whose activity is regulated by TFAM, TFB1M and TFB2M (Fig. **1**) [37, 38]. Translation of the mtDNA‐encoded genes into proteins requires the participation of specific nDNA-encoded factors such as initiation factor 2 (mtIF2) and 3 (mtIF3), translational release factor 1-like (mtRF1L), and recycling factors (mtRRF1 and mtRRF2) (Fig. **1**) [39]. mtDNA stability and damage response are controlled by TERT activity [40]. TERT can also lower mitochondrial ROS production and inhibit ROS-mediated apoptosis by increasing the intracellular glutathione (GSH)/glutathione disulfide (GSSG) ratio [41, 42]. Mitochondrial biogenesis requires the import of over 1000 mitochondrial proteins encoded in the nucleus and synthesized in the cytoplasm as precursor proteins (pre-proteins). These contain an amino‐terminal cleavable targeting signal for the recognition by specific mitochondrial surface receptors, and once inside, they are directed to their functional mitochondrial sub-compartments destination [43]. The translocase of the inner mitochondrial membrane 23 (TIM23) complex directs pre-proteins towards the mitochondrial matrix, where they are sorted to the intermembrane space or to the inner mitochondrial membrane (IMM) [44, 45]. The energy required for TIM23 channel activation and translocation into the matrix is provided by the mitochondrial membrane potential (Δψ) across the IMM and the oxidative phosphorylation-derived ATP [46, 47]. Several signals can activate mitochondrial biogenesis, but the AMP/ATP ratio and Ca^2+^ levels are the primary stimuli. Increased levels of AMP can trigger the PGC-1α/NRF-1/2-TFAM pathway through AMPK activation, which in turn directly phosphorylates PGC-1α (Fig. **1**) [48, 49]. Phosphorylated PGC-1α can then regulate the expression of several mitochondrial genes as well as its own expression [50, 51]. PGC-1α activation can also result from the activation of cAMP-PKA-CREB cascade. Specifically, AMP can be converted to cyclic AMP (cAMP) by adenyl cyclase (AC), which activates the cAMP-dependent kinase (PKA). This in turn phosphorylates the transcription factor cAMP response element binding protein (CREB) to promote PGC-1α expression, leading thus to activation of mitochondrial biogenesis [51, 52]. On the other hand, elevated Ca^2+^ levels can activate mitochondrial biogenesis by stimulating the calcium/calmodulin-dependent protein kinase (CaMK), which in turn phosphorylates p38 mitogen-activated protein kinase (MAPK), leading to the activation of PGC-1α, ultimately resulting in increased mitochondrial biogenesis (Fig. **1**) [23]. Furthermore, CaMK can also stimulate PGC-1α through CREB [23]. Finally, an increased NAD+/NADH ratio can also result in mitochondrial biogenesis activation through SIRT-1-mediated PGC-1α deacetylation (Fig. **1**) [53].

## 3 MITOCHONDRIAL BIOGENESIS MODULATION BY RESVERATROL IN NEURODEGENERATIVE DISEASES

Mitochondria are crucial for any obligate aerobic cell, including neurons, cells react to a low-energy state by either up or downregulating the mediators of mitochondrial biogenesis [54]. Any dysfunction of this cell mechanism can lead to severe diseases such as cancer, metabolic syndrome, and neurodegenerative diseases [55]. As a result, mitochondrial biogenesis is intensively investigated in the most common neurodegenerative diseases (Alzheimer’s disease, Parkinson’s disease, Huntington’s disease, cerebral stroke, *etc.*) that share the deregulated expression of effectors of mitochondrial biogenesis [12, 54]. Accordingly, activation of mitochondrial biogenesis is an important therapeutic target to either inhibit the progression of these diseases or improve recovery from brain injuries [56]. Various physiological factors (including fasting, exercise training, electric stimulation, and hormones) can activate mitochondrial biogenesis by acting on its effectors [55]. For instance, caloric restriction and exercise can, *in vitro* and in animal models, activate AMPK-SIRT1 signaling, increase mtDNA replication, and activate PGC-1α, NRF1, TFAM, and mitochondrial proteins in the cerebral cortex after cerebral ischemia [57, 58]. On the other hand, pharmacological factors, drugs, and polyphenols have been reported to activate mitochondrial biogenesis [59-62]. Within polyphenols, resveratrol displays neuroprotective properties in several neurodegenerative disorders, as well as a great ability to modulate mitochondrial biogenesis by acting on different key effectors (*e.g*., SIRT1, AMPK, PGC-1α, cAMP), through multiple mechanisms of action [55, 61, 63]. We next describe the impact of resveratrol on mitochondrial biogenesis modulatory machinery by analyzing and discussing its action on the main effectors of neuronal mitochondrial biogenesis.

### 3.1 PGC-1α

Peroxisome proliferator-activated receptor (PPAR)γ coactivator-1α (PGC-1α) is the master regulator of mitochondrial biogenesis [64], it was discovered as PPARγ transcriptional coactivator in several critical metabolic processes, such as in biological responses that require the shift from glycolytic to oxidative metabolism and in high energy-demand tissues like the muscle tissue [64, 65]. In this regard, PGC-1α can elevate myofibrillar protein expression, increase oxidative metabolism, and promote muscle fiber-switching into a type of higher mitochondrial density [66]. PGC-1α is also important in prolonging lifespan and protecting from metabolic diseases during aging, i, increased muscle PGC-1α protein expression was shown to promote several functions such as preserving mitochondrial activity, muscle integrity, neuromuscular junctions and inhibiting proteasomal degradation, autophagy, and apoptosis in transgenic mice [67]. On the other hand, knockout of PGC-1α in mice results in the loss of pigmented dopaminergic neurons, the hallmark of PD [68]. In addition, PGC-1α knockout mice displayed phenotypic characteristics and gene expression profiles similar to HD transgenic mice models [68]. In confirmation, the levels of PGC-1α and mitochondrial markers are reduced compared to control in tissues of human PD patients and are correlated with the severity of the disease [68]. Furthermore, PGC-1α lentiviral delivery has been shown to reduce neuronal loss and improve cognitive functions in the preclinical stages of AD patients [69]. Due to its central role in maintaining neuronal survival and synaptic transmission, PGC-1α activation or upregulation is a key therapeutic approach to counteract the development and progression of neuronal damage. Resveratrol administration has been reported to promote PGC-1α activity through the induction of AMPK and SIRT1, which are essential for increasing PGC1-α content [70, 71]. Specifically, resveratrol activates AMPK, which in turn activates PGC-1α in a SIRT1-dependent manner, for this reason, resveratrol is not able to exert its neuroprotective effect, in the absence of SIRT1 [71]. The ability of resveratrol to mediate PGC-1α activation has been reported in different physiological processes, for instance, resveratrol was found to elevate PGC-1α mRNA and protein levels and reduce oxidative stress in the lungs of a rat model of chronic obstructive pulmonary disease [72]. Hypoxia induction in a steroidogenic human ovarian granulosa-like tumor cell line (KGN) resulted in the downregulation of SIRT1, PGC-1α, and mtDNA, an effect that was reversed by resveratrol through SIRT1-mediated deacetylation and activation of PGC-1α [73]. Interestingly, resveratrol promotes mitochondrial biogenesis in hyperglycemic podocytes *in vitro via* the SIRT1-PGC-1α axis, while PGC-1α knockdown abolishes resveratrol's beneficial effect [74]. In a rat model of diabetes-induced cardiac myopathy, resveratrol was reported to increase mRNA and protein levels of both PGC-1α and NRF-1 and promote mitochondrial biogenesis, however, SIRT1 inhibition abolished this effect, confirming that the effect of resveratrol occurred through the SIRT-mediated deacetylation and activation of PGC-1α [75]. Accordingly, resveratrol induced the activation of oxidative phosphorylation and mitochondrial biogenesis genes, *in vitro* and in mice, through SIRT1-mediated deacetylation of PGC-1α, leading to an increase in PGC-1α activity [76]. Moreover, resveratrol was reported to promote skeletal muscle type switching to the slower, more oxidative phenotype *via* activation of SIRT1 and PGC-1α in a mouse model of Duchenne muscular dystrophy [77]. Finally, the SIRT3/FoxO3a pathway is another route used by resveratrol for enhancing transcriptional regulation of PGC-1α, as demonstrated by Fu *et al.* in mouse renal tubular TCMK-1 cells [78]. In the context of neurological disorders, Zhou *et al.* reported the protective effect of resveratrol in a preclinical model of early brain injury following subarachnoid hemorrhage (SAH), a fatal cerebrovascular disease [79]. Here, resveratrol improves mitochondrial biogenesis by increasing the protein expression of both PGC-1α and downstream transcriptional targets NRF-1 and TFAM [79]. Moreover, since PGC-1α is a powerful ROS scavenger, by promoting the expression of the ROS-detoxifying enzymes superoxide dismutase (SOD), resveratrol-induced mitochondrial biogenesis was able to eliminate ROS overproduction by mitochondria and recover brain tissue from ROS-induced damage [79-81]. Furthermore, resveratrol promoted mitochondrial biogenesis in a rat model of status epilepticus, by increasing protein expression of PGC-1α, NRF1, TFAM, as well as the mtDNA, and attenuation of neuronal cell damage in the hippocampus following status epilepticus [82]. Resveratrol-mediated enhancement of mitochondrial biogenesis mitigated the mitochondrial dysfunction associated with the pathogenesis of glaucomatous neurodegeneration *in vitro* [83]. Specifically, *in vitro* experiments performed in the RGC-5 retinal ganglion cell line, confirmed the SIRT1-dependent activation of PGC-1α following resveratrol application, indeed, knockdown of SIRT1 abolished the resveratrol-mediated PGC-1α activation and subcellular translocation [83]. Microglia are a type of glial cell located throughout the brain and spinal cord, they exert immune function in the central nervous system and are key players in the initiation of neurodegenerative diseases [84]. Microglia display a dual phenotype: proinflammatory (M1) and anti-inflammatory (M2). M1-inhibition and M2-stimulation have been suggested as potential therapeutic approaches for treatment of neuroinflammation-related diseases [84, 85]. In this regard, resveratrol was shown to reduce inflammatory damage by regulating microglia M1/M2 polarization *via* PGC-1α, indeed, resveratrol-induced PGC-1α-overexpression promoted M2 polarization *in vitro* and in a mouse model of neuroinflammation. On the other hand PGC-1α- knockdown attenuated M2 polarization *in vitro* [85].

**Fig. (2):**
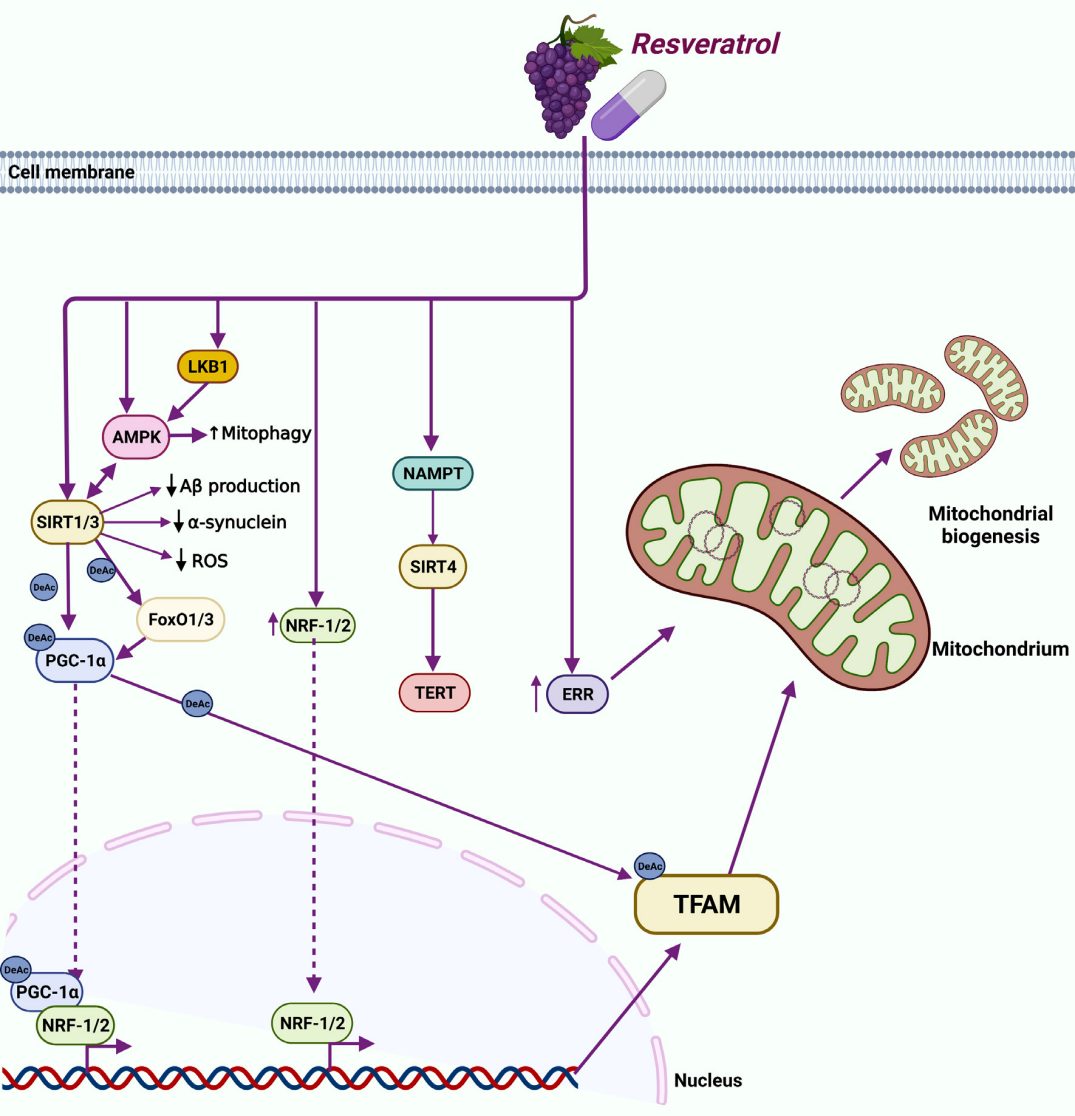
Overview of resveratrol-modulated mitochondrial biogenesis in neurodegenerative diseases. PGC-1α: resveratrol-induced mitochondrial biogenesis in neurodegenerative disorders can take place by either SIRT1/PGC-1α/NRF-1 or SIRT1/FoxO3a/PGC-1α pathways, where resveratrol activates PGC-1α in a SIRT1-dependent manner. NRF-2: resveratrol increases NRF-2 protein levels and promotes NRF-2 nuclear translocation. SIRTs: resveratrol-activated SIRT1 can protect neurons by reducing ROS accumulation, Aβ oligomer formation, and α-synuclein accumulation. In addition, resveratrol-activated SIRT1 restores mitochondrial function by increasing PGC-1α and TFAM expression. AMPK: in neurons, resveratrol-stimulated AMPK activity requires activation by either SIRT1 or the upstream activator LKB1. Moreover, the resveratrol-activated AMPK/mitophagy pathway alleviates mitochondrial network fragmentation and dysfunction. ERRα: resveratrol enhances mitochondrial biogenesis by increasing ERRα expression and activity. TERT: resveratrol-mediated activation of TERT depends on nicotinamide phosphoribosyltransferase (NAMPT) activation, which activates SIRT4. TFAM: mitochondrial biogenesis-associated RES-elicited neuroprotection acts *via* the SIRT/PGC-1α signaling pathway, ultimately affecting TFAM acetylation rate. Dashed lines are for translocation.

Overall, resveratrol-induced mitochondrial biogenesis occurs through the SIRT1/PGC-1α/NRF-1 and SIRT1/FoxO3a/ PGC-1α pathways (Fig. **2**), leading to beneficial outcomes in neurodegenerative disorders and other diseases. Nonetheless, these findings need to be translated from the bench to the bedside by performing well-designed clinical trials.

#### 3.1.1 NRF-1 and NRF-2

NRF1 and NRF2 act downstream of PGC-1α to regulate mtDNA transcription and replication, mainly by controlling TFAM, TFB1M and TFB2M, as well as other mitochondrial processes such as the oxidative phosphorylation [29-32, 86]. For example, NRF1 and NRF2 can regulate the expression of nuclear-encoded mitochondrial electron transfer chain (ETC) proteins [87]. In particular, NRF2 regulates the expression of the nuclear-encoded subunit of the mitochondrial translocation complex, mitochondrial enzyme translocase outer mitochondrial membrane (TOMM20), a key enzyme involved in mitochondrial membrane transport [88]. NRF-1 is also involved in importing the nuclear-encoded precursor proteins into the mitochondria [89]. In response to oxidative stress and inflammation, NRF-2 translocates from the cytoplasm into the nucleus and activates the transcription of genes with antioxidant activity [90]. Despite this mechanism, oxidative stress is the leading player in neurodegenerative diseases development and progression, accordingly, NRF-2 levels have been found to be reduced in HD transgenic mice models, as well as in autopsy of brain tissues from patients with PD and AD [91, 92]. Moreover, NRF2 induction was shown to ameliorate cognitive impairment in AD animal models by suppressing oxidative stress and neuroinflammation. [93, 94]. Similarly, lower levels of NRF-1 have been reported in PD and AD patients [95, 96], while in HD, the age of clinical onset of the disease is associated with polymorphisms in the NRF-1 gene [97]. Several studies pointed out NRF-1 and NRF-2 as targets of resveratrol for enhancement of mitochondrial biogenesis. Yang *et al.* reported that resveratrol treatment increased neuronal viability and inhibited neuronal apoptosis *in vitro*, besides, the resveratrol protective action was exerted by increasing NRF-2 protein expression and promoting nuclear translocation of NRF-2 [98]. A similar effect was also reported *in vivo*, where resveratrol administration in rats, following ischemia/reperfusion-induced cerebral damage, increased nuclear NRF-2 protein levels, suggesting a role of resveratrol in promoting NRF-2 nuclear translocation (Fig. **2**) [99]. Furthermore, resveratrol was shown to ameliorate oxidative damage, increase activation and nuclear localization of transcription factor Nrf-2 and preserve mitochondrial function in neurons of a spinal cord hypoxic injury rat model (Fig. **2**) [100]. Finally, resveratrol was able to revert the sodium fluoride-induced neurotoxicity in a rat model as well as restore the suppression of NRF-2 and improve mitochondrial biogenesis in a SIRT1-PGC-1α-dependent manner both *in vitro* and *in vivo* (Fig. **2**) [101]. With regard to NRF-1, administration of a micronized preparation of resveratrol, SRT501-M, resulted in neuroprotection of the cerebral cortex of HD transgenic mice *via* the enhanced expression of PGC-1 and NRF-1 [102]. Although not in the context of neurological disorders, resveratrol's ability to increase NRF-1 levels and improve mitochondrial content has also been confirmed in human umbilical vein endothelial cells, where resveratrol overcomes diesel exhaust particulate extracts-induced inhibition of NRF-1 [103]. Furthermore, two months of oral administration of resveratrol significantly improved the gene expression of NRF-1, SIRT1, PGC-1α, PPAR-α, and TFAM, as well as the mtDNA content in cardiac tissue of estrogen-deficient female rats [104].

### 3.2 SIRT1

SIRT1 belongs to the sirtuins protein family, which is part of class III histone deacetylases that require one molecule of NAD^+^ for each deacetylation cycle [105]. SIRT1 is present in both the nucleus and the cytoplasm [105]. Nuclear SIRT1 deacetylates various transcription factors, thereby enhancing the expression of several metabolic and cell cycle genes [105]. In the liver, SIRT1 regulates and increases the gluconeogenic/glycolytic pathways in response to fasting by PGC-1α and FoXO1 deacetylation [106]. In contrast, during late fasting, the presence of SIRT1 deacetylates CRCT2 facilitating its degradation [107]. SIRT1 also interferes in fatty acid oxidation and synthesis in response to starvation and fasting. SIRT1 stimulates fatty acid oxidation by activating the nuclear receptor, peroxisome proliferator-activated receptor-α (PPARα), which is essential for the activation of PGC-1α [108]. Because of their roles in age-related diseases and their involvement in biological pathways (stress response, mitochondrial dysfunction, oxidative stress, *etc*.) associated with age-related neurodegenerative disorders, sirtuins, especially SIRT1, have attracted great interest in recent years [109, 110]. SIRT1 is primarily expressed in neurons and in the adult brain, with high levels in the cortex, hippocampus, cerebellum and hypothalamus [111], besides, SIRT1 is the most extensively studied sirtuin in the context of neurodegenerative diseases such as AD, PD, and HD [109, 110]. In AD, SIRT1 levels in healthy individuals decline during aging, increasing the risk of developing AD, besides. In agreement, SIRT1 expression levels were lower in the brain of a mouse model of AD compared to the control group [110, 112]. SIRT1 protective function has also been observed *in vitro*, in animal models of PD, and to some extent in human AD patients, where aggregation of α-synuclein protein mediated a decrease of SIRT1 expression, regulation of apoptosis, and mitochondrial dysregulation, leading to PD development [110, 113]. In HD, SIRT1 was reported to have protective effects against the neurotoxicity of the mutant huntingtin (mHTT) protein, the leading cause of HD, besides, SIRT1 overexpression in a transgenic mouse model of HD showed neuroprotective effects by reducing ROS production [110, 114, 115]. Plenty of evidence indicates that resveratrol improves mitochondrial function *via* SIRT1 activation (either directly or indirectly), which then exerts its deacetylase activity through different mechanisms [71, 116, 117]. For instance, SIRT1 can induce PGC-1α expression and activity once activated, provoking LKB1 deacetylation and activation, which then regulates AMPK [70, 118]. Resveratrol-induced increase in nuclear SIRT1 levels can also improve the expression of manganese superoxide dismutase (MnSOD) and reduce oxidative stress in cardiomyocytes [119]. Moreover, regulation of antioxidant genes by activated SIRT1 can also depend on FoxO3a/PGC-1α complex formation (Fig. **2**) [120]. In the context of neurodegenerative diseases, a plethora of evidence confirmed SIRT1-mediated neuroprotective effects of resveratrol. For instance, in AD, resveratrol-activated SIRT1 was suggested to deacetylate and repress p53 activity, preventing apoptotic cell death of neurons (Table **1**) [121]. Besides, resveratrol-activated SIRT1 can protect neurons by reducing ROS accumulation and Aβ oligomer formation in brains of AD patients [121] (Fig. **2**). This antioxidant mechanism-linked neuroprotective property of resveratrol is promoted through both inhibition of prooxidative factors (NADPH and iNOS) [122], and enhanced expression of antioxidant enzymes such as SOD, catalase, thioredoxin, and glutathione peroxidase (GPx) [123]. In addition, resveratrol can enhance cognitive function and induce neuroprotection in a transgenic mouse model of AD by improving proteostasis to prevent the accumulation of aberrant amyloid and tau proteins (Table **1**) [124]. In the same study, resveratrol increased the levels of neprilysin, a main amyloid-degrading enzyme [124]. Along the same lines, resveratrol reduced the activity of β-secretase (BACE1), essential for the production of Aβ amyloid species, through the activation of SIRT1 in rodent models of AD (Table **1**) [125, 126], confirming its ability to reduce the amyloidogenic pathway. Furthermore, it was reported that resveratrol can induce neuroprotection by activating SIRT1 to inhibit autophagy, in a mouse model of Pb-induced neurotoxicity [127]. Similarly, resveratrol reversed the reduction in SIRT1 expression and recovered neurons from Aβ1-42-induced defects in spatial learning, synaptic plasticity, and memory in a rat model of AD (Table **1**) [128]. Recently, selenium-based nanoformulations of resveratrol (RSV-SeNPs) were shown to mitigate oxidative stress, neuroinflammation, and mitochondrial dysfunction by upregulating the expression of SIRT1, Aβ peptides and decreasing tau hyperphosphorylation, and reducing the levels of microRNA-134 in aluminum chloride-induced AD in rats. As such, RSV-SeNPs increased neurite outgrowth and improved neurocognitive functions in this animal model (Table **1**) [129]. Other studies indicated that the anti-inflammatory mechanism-linked neuroprotective property of resveratrol contributes to protection from brain damage. For instance, resveratrol treatment, post-induction of ischemia in rodent models, activates hippocampus astrocytes and microglia and suppresses the inflammatory response mediated by nuclear factor κ-light-chain-enhancer of activated B cells (NF-κB), cyclooxygenase-2 (COX-2), and nitric oxide synthase (NOS) in rat hippocampal cells [130]. One clinical study reported that resveratrol decreases Aβ levels in cerebrospinal fluid, attenuates neuroinflammation, and induces adaptive immunity in mild to moderate AD patients [131]. Anton *et al.* carried out a pilot trial in the elderly population and found that chronic use of resveratrol for 90 days can improve psychomotor speed without enhancing other cognitive functions like memory (Table **1**) [132]. In addition, Wang *et al.* reported that resveratrol administration can act as an AD-adjuvant therapy to help the engraftment of human mesenchymal stem cells *via* the SIRT1-mediated overexpression of neurotrophic factors associated with enhanced neurogenesis, neuron survival, learning, and memory in a mouse model of AD (Table **1**) [133]. PD pathogenesis is linked to α-synuclein protein misfolding and aggregation in neuronal cells [134]. In this regard, resveratrol-activated SIRT1 was shown to reduce α-synuclein accumulation (Fig. **2**) by regulating autophagy and heat shock factor 1(HSF1) deacetylation level in cells overexpressing α-synuclein *in vitro* [113]. Moreover, neuronal protection against apoptosis and cell death *via* resveratrol-induced SIRT1 activity has also been demonstrated in PD *in vitro* and *in vivo* studies [113]. Additionally, *in vitro* studies and animal models of PD have demonstrated that resveratrol treatment-induced SIRT1 activity leads to protecting neurons against cell death [113]. Resveratrol also had a neuroprotective effect *in vivo* in 6-OHDA-induced PD in rats by reducing the levels of inflammatory markers [135]. A recent study demonstrated that resveratrol could rescue the oxidative stress induced by loss of function of parkin in Drosophila melanogaster (Table **1**) [136]. Finally, in animal models of HD, resveratrol-activated SIRT1 restored mitochondrial function and elevated PGC-1α and TFAM protein levels (Fig. **2**) [137].

### 3.3 AMPK

The enzyme AMPK is considered the guardian of metabolism and mitochondrial homeostasis, indeed, it is activated when ATP synthesis is impeded or when ATP is being utilized at a high rate, resulting in the increased generation of AMP [50, 138]. In low energy conditions, AMPK phosphorylates specific enzymes to both increase ATP generation and decrease ATP consumption, it also phosphorylates other proteins involved in various signaling pathways, including mTOR complex 1 (mTORC1), lipid homeostasis, glycolysis, and mitochondrial homeostasis [139]. AMPK is an essential mitochondrial biogenesis activator, and key players of this process, such as PGC-1α and SIRT1, are phosphorylated and triggered by AMPK [138, 139]. Deregulation of AMPK has been linked to several neurodegenerative diseases, including multiple sclerosis, amyotrophic lateral sclerosis, AD, HD, and PD, but the mechanisms underlying this association are still unclear [140]. AMPK-knockout in mice did not show any impact on brain development in embryonic stages but rather in the postnatal and aging brain. Moreover, depending on the neuronal cell type, type of insult, intensity and duration of activation, AMPK effects can be either neuroprotective or pro-apoptotic [141]. Resveratrol's ability to alleviate oxidative stress-induced damages *via* AMPK activation has been observed in different cells and tissues. Indeed, resveratrol improves the survival of heart muscle cells by modulating the AMPK signaling pathway in H_2_O_2_-induced ischemic conditions, and inhibition of AMPK causes the loss of the observed protective effect, confirming resveratrol action is AMPK-mediated [142]. In skeletal muscle cells and neuronal cells *in vitro*, resveratrol ameliorates both insulin resistance and glucose uptake, respectively, *via* AMPK activation [143, 144]. Moreover, resveratrol stimulates the AMPK-SIRT1-PGC-1α axis, preventing the accumulation of renal lipids and cell injury in an animal model of renal nephropathy [145]. With regard to mitochondrial biogenesis, Kang *et al.* [146] showed that resveratrol alleviates oxidative stress-induced mitochondrial dysfunction caused by treatment with benzo(a) pyrene (BaP) in primary neurons. Specifically, resveratrol alleviates BaP-indued mitochondrial network fragmentation by both enhancing mitochondrial biogenesis *via* the AMPK/PGC-1α pathway and activating mitophagy through the PINK1-Parkin and AMPK/ULK1 pathways, thereby coordinating mitochondrial homeostasis (Fig. **2**) [146]. In SH-SY5Y neural cells, resveratrol rescues ischemic stroke-induced oxygen glucose deprivation (OGD), cell death and mitochondrial dysfunction *via* AMPK activation [147]. Neuronal activation of AMPK and the AMPK-dependent mitochondrial biogenesis stimulation by resveratrol have also been reported in the study of Dasgupta *et al.* [148]. Here, authors indicate that resveratrol-stimulated AMPK activity in neurons depends on the tumor suppressor LKB1 activity (an upstream kinase activator of AMPK) but does not require SIRT1(Fig. **2**) [148]. However, in cellular models of PD, resveratrol requires both AMPK and SIRT1, specifically, resveratrol neuroprotection is exerted *via* AMPK/SIRT1/ mitophagy pathway induction (Fig. **2**) [149]. Resveratrol can partially rescue mitochondrial dysfunction in cultured human fibroblasts mutant for *Parkin 2*, whose mutation is responsible for a form of early-onset parkinsonism in humans. Here, resveratrol could enhance mitochondrial respiration through the AMPK/SIRT1/PGC-1α axis (Table **1**) [150]. Besides, suppression of AMPK causes SIRT1 inhibition and attenuates resveratrol-mediated protective effects on rotenone-induced apoptosis *in vitro* [149]. Resveratrol-activated AMPK/ mitophagy pathway has also been reported in a rat model of ischemic brain injury where resveratrol increases phosphorylated AMPK levels in the cerebral cortex of rats subjected to middle cerebral artery occlusion (MCAO) [151]. Moreover, resveratrol reduces superoxide anion production in neuronal cultures exposed to glutamate-induced excitotoxicity, prevents the overload of intracellular Ca^2+^ associated with mitochondrial failure, and reduces cell death [151]. Furthermore, in both models, resveratrol promotes mitophagy (Fig. **2**) [151]. A recent study performed in a cell model of AD showed that Aβ production increases in a resveratrol dose- and time-dependent manner *via* stabilization of amyloid precursor protein (APP), and AMPK-mediated inhibition of trypsin-like proteasome activity [152]. Precisely, an intermediate dose of resveratrol treatment for 24 h unexpectedly increases Aβ production, while higher concentrations or shorter treatment durations do not provide the same result, conversely, high doses of resveratrol decrease Aβ secretion and β-secretase activity at any treatment duration [152]. It has also been shown that in neuronal and non-neuronal cells *in vitro* and in mice models of AD, AMPK mediates resveratrol’s anti-amyloidogenic effects (Table **1**) [153, 154]. Moreover, intracerebroventricular injection of resveratrol alleviated Aβ-induced learning and cognitive defects in a mouse model of AD. This was accompanied by upregulation of AMPK/ PGC-1α and downregulation of markers of inflammation like NF-κB/IL-1β/NLRP3 (Table **1**) [155]. Several studies reported that resveratrol's effects are closely dependent on the dosage, unlike the observation by Shaito *et al.,* who reported that beneficial effects are mainly associated with a low dosage of resveratrol [156-160]. For instance, resveratrol acts as an antioxidant and anti-apoptotic factor, improving cell viability, at low doses, *vice versa* at high doses, it acts as a pro-oxidant and pro-apoptotic, decreasing cell viability [156-160]. Therefore, dose dependency is a crucial aspect to be considered when therapeutic employment of resveratrol is being planned. In the context of neurodegenerative diseases, the AMPK-mediated resveratrol-enhanced mitochondrial biogenesis is overall well established, although clinical studies require well-designed protocols using the appropriate dosages and treatment durations of resveratrol.

### 3.4 ERRs

The expression of important mitochondrial biogenesis-associated genes is under the control of both the PGC-1 family of transcriptional coactivators and the nuclear receptors subfamily of estrogen-related receptors (ERRs) [161]. ERR-α, ERR-β, and ERR-γ belong to this subfamily [161]. Interestingly, mice null for ERRs and mice lacking PGC-1α have some phenotypes in common, such as the decrease in mitochondrial gene expression, development of heart failure, and inability to adapt their body’s temperature when exposed to a cold environment [162]. In addition, ERRs enhance the expression of PGC-1α and activate NRF transcription factors [161]. ERRα plays a crucial role in AD pathology and may function as a potential therapeutic target. Indeed, overexpression of ERRα was shown to attenuate both Aβ extracellular accumulation and Tau proteins hyperphosphorylation, critical AD pathogenic effectors causing neurofibrillary tangles (NFTs) and neuronal loss [163]. In PD, parkin protein was shown to bind ERRs and increase their ubiquitination and degradation *in vitro* using cultured neuronal cell lines and in a mouse model of PD [164], on the other hand, parkin-mediated degradation of ERR is attenuated in PD patients’ fibroblasts with mutated parkin and parkin-null mice [164]. Furthermore, ERRα mRNA levels are significantly suppressed in an HD mouse model compared to wild type [165]. In the same study, ERR mRNA levels were found to increase in the cerebral cortex of wild type mice undergoing therapeutic treatment with the creatine analog guanidinopropionic acid, but not in HD mice treated with the same molecule [165]. Resveratrol was also shown to enhance ETC protein complexes, oxygen consumption, and mitochondrial biogenesis through activation of ERRα in fibroblasts of patients with deficiency in the ETC proteins (Fig. **2**) [166, 167]. Specifically, resveratrol treatment enhances ERRα expression, attenuates diet-induced cardiac hypertrophy and stimulates mitochondrial activity in mice fed a high-fat diet [168]. Consistently, ERRα silencing abolishes resveratrol benefits and reduces mitochondrial functions (ATP production, oxygen consumption, and complex I activity), confirming the critical role of ERRα in mediating resveratrol-induced cardiac protection [168]. Studies linking resveratrol signaling to ERRs in the process of mitochondrial biogenesis are limited and even scarcer in relation to neurodegenerative diseases, warranting future investigation into this area.

### 3.5 TERT

The primary function of TERT is ensuring the chromosomes' stability by maintaining telomere length, which allows cells to evade senescence [169]. TERT can also regulate the expression of genes involved in immune responses, inflammation, and cell differentiation, including NF-κB and wingless/integrated (Wnt)/β catenin signaling pathway genes [169]. TERT is commonly found in the cytoplasm, however, it can translocate into cellular compartments like the nucleus and the mitochondria, which contain about 20% of the total cellular TERT [170]. TERT binds the mtDNA and protects it from environmental damage [171], it also protects mitochondria by modulating mtDNA damage signals and reducing mitochondrial ROS production, thereby improving ETC activity [41, 42, 170]. TERT was found to inhibit ROS-mediated apoptosis by increasing the intracellular GSH/GSSG ratio [41, 42]. Neurons of human brain tissues lack TERT activity due to the downregulation of its RNA subunit hTERT. However, TERT might also have some non-canonical functions since hTERT downregulation has also been observed at very early developmental stages, in addition, TERT protein is maintained without any telomerase activity in activated microglia and in the neurons of the adult human brain [172, 173]. Although the exact role of TERT in the brain is still unclear, it has been suggested that it may provide protection against oxidative damage [172]. This is supported by the finding that in cultured neurons from TERT knockout mice, neurons rich in hyperphosphorylated tau exhibit higher levels of oxidative stress [172]. In addition, hyperphosphorylated tau levels are negatively correlated with TERT protein levels [172]. Interestingly, TERT protein accumulates in the mitochondria during AD development, however, it remains unclear whether its translocation to the mitochondria is a response to oxidative stress or is part of TERT's neuroprotective function [172]. Besides, telomerase activators increased TERT levels in neurons and exerted protection against neuronal damage *in vitro* and in mice models of AD [173]. Resveratrol was shown to increase TERT and telomerase activity in various cells and tissues, for instance, it activates TERT in human aortic smooth muscle cells, and increases telomerase activity in mouse heart and liver tissues, supporting the anti-aging effect of TERT in these cells [174]. Here, the resveratrol action is exerted *via* the NAMPT-SIRT4-hTERT axis, indeed, resveratrol-mediated activation of TERT is dependent on nicotinamide phosphoribosyltransferase (NAMPT) activation, which in turn activates SIRT4 (Fig. **2**) [174]. Resveratrol also augments telomerase activity and increases TERT phosphorylation in endothelial progenitor cells, delaying the onset of their senescence [175]. An opposite effect has been observed in different cancer cell lines where resveratrol reduces TERT and induces cellular apoptosis [176, 177]. Resveratrol also inhibits the expression of TERT in metastatic glioblastom cancer cells, a phenomenon associated with dose-dependent prevention of cell proliferation and tumor regression [178]. Data on resveratrol-enhanced mitochondrial biogenesis through TERT are still limited, especially regarding neurodegenerative diseases. However, mechanisms of resveratrol-mediated protection similar to those discovered in other cells and tissue may be conceivable in neuronal cells. Indeed, this area remains an area of intense exploration, hoping to uncover new therapeutic targets for neurodegenerative diseases.

### 3.6 TFAM

TFAM is part of the PGC-1α/NRF-1-2/TFAM signaling pathway, the main mitochondrial biogenesis regulatory pathway [9]. Several studies report that resveratrol can modulate mitochondrial biogenesis in pathological neurons by regulating the expression of this transcription factor. In a rat model of brain injury induced by subarachnoid hemorrhage (SAH), resveratrol was able to significantly upregulate the protein levels of signaling molecules acting downstream of PGC-1α like TFAM [79]. The final effect of resveratrol administration was the reduction of SAH-induced brain edema, along with the amelioration of apoptosis, and restoration of mitochondrial membrane potential and ATP levels [79]. Overall, the results from this study pointed out that resveratrol exerts neuroprotection in a model of brain injury by promoting mitochondrial biogenesis and function *via* the activation of PGC-1α and its downstream signaling molecules [79]. Resveratrol was also reported to be able to exert neuroprotection in a manganese (Mn)-induced model of brain injury in cultured primary neurons from mice. Data from this study demonstrated resveratrol’s ability to counteract ROS increase, MMP reduction, and the associated Mn-elicited mitochondrial dysfunction. This resveratrol-mediated phenomenon was in part due to SIRT3-mediated TFAM deacetylation (Fig. **2**) [179]. Similar to Mn, sodium fluoride (NaF) was reported to cause mitochondrial dysfunction *in vitro* and *in vivo* and impair mitochondrial biogenesis by inhibiting SIRT1 deacetylase activity and decreasing PGC-1α, NRF1, and TFAM protein levels. The ability of resveratrol to ameliorate NaF-induced neuronal damage involved SIRT1-dependent activation of PGC-1α/NRF1/TFAM signals and an increase of the protein levels of all the signaling pathway components (Fig. **2**) [79, 180]. In an *in vivo* rodent model of kainic acid-induced seizure, resveratrol attenuated epilepsy-induced neuronal apoptosis and increased the expression of the mitochondrial biogenesis modulators PGC-1α, NRF1, and TFAM. In this case, data also indicated the potent neuroprotective effects of resveratrol and further highlighted its capability to affect PGC-1α downstream molecules, including TFAM, leading to enhanced mitochondrial functionality and neuronal protection [82]. In a cell model of HD, resveratrol reversed the disease-associated PGC-1α and TFAM protein decrement, restoring the lost mitochondrial membrane potential and rescuing mitochondrial function [137]. Consonant with the *in vitro* findings, 24 days of resveratrol administration to YAC128 mice, a model of HD, significantly improved both learning activity and motor coordination by augmenting the gene expression of ETC-associated proteins [137]. Overall, the presented data strongly indicate that resveratrol-elicited neuroprotection in neurodegenerative conditions is associated with an improvement of mitochondrial biogenesis/function due to activation/ overexpression of PGC-1α/NRF1/TFAM pathway components (Fig. **2**).

## CONCLUSION

Over the past years, an ever-growing number of studies have indicated that naturally occurring polyphenol resveratrol can provide protection in several neurodegenerative conditions. Interestingly, besides acting through its well-known antioxidant and anti-inflammatory properties, resveratrol can provide neuroprotection by impacting the process of mitochondrial biogenesis, a phenomenon of paramount importance for maintaining proper neuronal plasticity and function. The discovery that resveratrol can interact with several important mitochondrial biogenesis effectors could pave the way to identify new druggable targets and markers of onset and progression of neurodegenerative conditions. Indeed, this peculiar action that resveratrol plays on specific mitochondrial biogenesis effectors might be exploited to develop a preventive treatment to counteract/delay age-related neurodegenerative diseases. It can also have applications in neurological conditions with limited therapeutic options where new therapeutic strategies are urgently needed.

Despite the promising results of the studies discussed in this review, further research is needed to confirm the potential therapeutic effects of resveratrol in animal models and human trials of neurodegenerative diseases. Building on these findings, future studies should employ different resveratrol concentration ranges, routes of administration and treatment durations, and rules of patient selection. This is of particular importance in light of resveratrol's complex pharmacokinetics and the delicate balance between its beneficial and adverse effects. In fact, besides the limitation of its low bioavailability, resveratrol dosage and the redox environment in the location of its application appear to have primary importance in determining the final outcome, whether neurotoxic or neuroprotective [156, 181]. Moreover, the ability of resveratrol to modulate disease-associated non-coding RNA (ncRNA) deserves deeper investigations as it may result in new therapeutic approaches against neurodegenerative diseases and other pathological conditions [182].

## Figures and Tables

**Table 1:** Studies showing the protective roles of resveratrol in Alzheimer's disease and Parkinson's disease using different experimental models.

**Disease/Model**	**Study Modality**	**Effect of Resveratrol**	**References**
AD	Clinical study	- Resveratrol-activated SIRT1 deacetylated p53, repressing its activity and preventing apoptotic cell death of neurons.	[121]
AD	AD transgenic mouse model (3xTg-AD)	- Resveratrol reduced levels of Aβ and p-tau proteins in 3xTg-AD mice. - Resveratrol reduced the levels of amyloidogenic secretase BACE1. - Resveratrol increased AMPK protein levels, and upregulated SIRT1 pathway, including activation of PGC-1α and CREB. - Resveratrol enhanced cognitive function and induced neuroprotection by improving proteostasis to prevent the accumulation of aberrant amyloid and tau proteins.	[124]
AD	Pb-induced oxidative damage and cognitive impairment in mice	- Resveratrol increased nuclear localization and phosphorylation of SIRT1 and increased protein levels of AMPK and PGC-1α. - Resveratrol repressed the Pb-induce amyloidogenic processing decreasing cortical Aβ1-40 levels. - Resveratrol ameliorated spatial learning and memory deficits.	[125]
AD	Amyloid-β protein precursor/presenilin1 (AβPP/PS1) mouse model	- Resveratrol activated SIRT1 and AMPK. - Reduced the amyloid burden and increased mitochondrial complex IV protein levels in mouse brain. - Resveratrol prevented memory loss.	[126]
AD	Amyloid-β protein precursor/presenilin1 (AβPP/PS1) mouse model	- Resveratrol reduced β-amyloid (Aβ) levels. - Resveratrol treatment or SIRT1 activation inhibited autophagy by inhibition of LC3 and Beclin-1 expression.	[127]
AD	Rat model of AD (Aβ1-42 injection into the hippocampus).	- Resveratrol reversed the reduction in SIRT1 expression. - Resveratrol reversed the reduction in CREB phosphorylation. - Resveratrol recovered neurons from Aβ1–42-induced defects in spatial learning, synaptic plasticity, and memory.	[128]
AD	Aluminum chloride-induced AD rat model	- Selenium-based nanoparticles of resveratrol (RSV-SeNPs) mitigate oxidative stress, neuroinflammation, and mitochondrial dysfunction. - RSV-SeNPs upregulate the expression of SIRT1, clear Aβ peptides, and decrease tau hyperphosphorylation. - RSV-SeNPs reduce the levels of microRNA-134 increasing neurite outgrowth. - RSV-SeNPs improve neurocognitive functions.	[129]
PD	Engraftment of human mesenchymal stem cells in a mouse model of AD.	- Resveratrol administration can act as an AD-adjuvant therapy to help the engraftment of human mesenchymal stem cells. - Resveratrol action is mediated *via* SIRT1. - SIRT1 mediates overexpression of neurotrophic factors leading to enhanced neurogenesis, neuron survival, learning, and memory.	[130, 133]
AD	Clinical study	- Resveratrol decreases Aβ levels in cerebrospinal fluid, attenuates neuroinflammation, and induces adaptive immunity in mild to moderate AD patients.	[131]
AD	Pilot clinical trial	- Chronic use of resveratrol for 90 days can improve psychomotor speed without enhancing other cognitive functions like memory.	[132]
PD	Rat model (6-OHDA-induced PD)	- Resveratrol treatment decreased the levels of COX-2 mRNA and protein. - Resveratrol treatment also decreased the levels of COX-2 mRNA TNF-α mRNA. - Resveratrol alleviated 6-OHDA-induced chromatin condensation and mitochondrial tumefaction of dopaminergic neurons	[136]
PD	*D. melanogaster* (parkin mutant)	- Resveratrol protected against decreased activities of acetylcholinesterase and catalase in parkin-mutant flies. - Resveratrol mitigated the accumulation of oxidative species hydrogen peroxide, nitric oxide and malondialdehyde in parkin-mutant flies. - Resveratrol alleviated locomotor deficits associated with loss of parkin function.	[137]
PD	Cellular models of PD	- In cellular models of PD, resveratrol protected against rotenone-induced apoptosis of SH-SY5Y cells and enhanced degradation of α-synuclein in α-synuclein-expressing PC12 cell lines by induction of autophagy, an AMPK/SIRT1 dependent manner.	[151]
PD	*In vitro* (Human fibroblasts mutant for *Parkin 2*)	- Resveratrol can enhance mitochondrial respiration through activation of the AMPK/SIRT1/PGC-1α axis.	[152]
AD	Neuronal and non-neuronal cells in culture and mouse models	- Resveratrol activates AMPK. - Resveratrol is neuroprotective by decreasing the amyloid proteins and tau hyperphosphorylation. - Resveratrol reduces cognitive impairment in mice.	[155, 156]
AD	Mouse model of AD (Aβ1-42 injection into the brain)	- Intracerebroventricular injection of resveratrol upregulated AMPK/ PGC-1α. - Resveratrol downregulation of markers of inflammation like NF-κB/ IL-1β/ NLRP3. - Resveratrol alleviated Aβ-induced learning and cognitive defects.	[157]

## LIST OF ABBREVIATIONS

AD: Alzheimer's Disease
ALS: Amyotrophic Lateral Sclerosis
AMPK: Adenosine Monophosphate Protein Kinase
APP: Amyloid Precursor Protein
ERR‐α: Estrogen‐related Receptor‐α
ERRs: Estrogen-related Receptors
ETC: Electron Transfer Chain
GPx: Glutathione Peroxidase
HD: Huntington’s Disease
HSF1: Heat Shock Factor 1
MCAO: Middle Cerebral Artery Occlusion
MnSOD: Manganese Superoxide Dismutase
NFTs: Neurofibrillary Tangles
OGD: Oxygen Glucose Deprivation
PD: Parkinson's Disease
PPARα: Peroxisome Proliferator-activated Receptor-α
ROS: Reactive Oxygen Species
SOD: Superoxide Dismutase
TERT: Telomerase Reverse Transcriptase
TFAM: Transcription Factor A Mitochondrial

## References

[1] Williams A. (2002). Defining neurodegenerative diseases.. BMJ.

[2] Erkkinen M.G., Kim M.O., Geschwind M.D. (2018). Clinical neurology and epidemiology of the major neurodegenerative diseases.. Cold Spring Harb. Perspect. Biol..

[3] Gerovska D., Araúzo-Bravo M.J. (2022). The common incidence-age multistep model of neurodegenerative diseases revisited: wider general age range of incidence corresponds to fewer disease steps.. Cell Biosci..

[4] Pereira T.M.C., Côco L.Z., Ton A.M.M., Meyrelles S.S., Campos-Toimil M., Campagnaro B.P., Vasquez E.C. (2021). The emerging scenario of the gut-brain axis: The therapeutic actions of the new actor kefir against neurodegenerative diseases.. Antioxidants.

[5] Singh A., Kukreti R., Saso L., Kukreti S. (2019). Oxidative stress: a key modulator in neurodegenerative diseases.. Molecules.

[6] Stephenson J., Nutma E., van der Valk P., Amor S. (2018). Inflammation in CNS neurodegenerative diseases.. Immunology.

[7] Lin M.T., Beal M.F. (2006). Mitochondrial dysfunction and oxidative stress in neurodegenerative diseases.. Nature.

[8] Wang C., Youle R.J. (2009). The role of mitochondria in apoptosis.. Annu. Rev. Genet..

[9] Golpich M., Amini E., Mohamed Z., Azman A.R., Mohamed I.N., Ahmadiani A. (2017). Mitochondrial dysfunction and biogenesis in neurodegenerative diseases: pathogenesis and treatment.. CNS Neurosci. Ther..

[10] Okamoto K., Kondo-Okamoto N. (2012). Mitochondria and autophagy: Critical interplay between the two homeostats.. Biochim. Biophys. Acta, Gen. Subj..

[11] Liu Y.J., McIntyre R.L., Janssens G.E., Houtkooper R.H. (2020). Mitochondrial fission and fusion: A dynamic role in aging and potential target for age-related disease.. Mech. Ageing Dev..

[12] Gao J., Wang L., Liu J., Xie F., Su B., Wang X. (2017). Abnormalities of mitochondrial dynamics in neurodegenerative diseases.. Antioxidants.

[13] Youle R.J., van der Bliek A.M. (2012). Mitochondrial fission, fusion, and stress.. Science.

[14] Zemirli N., Morel E., Molino D. (2018). Mitochondrial dynamics in basal and stressful conditions.. Int. J. Mol. Sci..

[15] Sanchis-Gomar F., García-Giménez J., Gómez-Cabrera M., Pallardó F. (2014). Mitochondrial biogenesis in health and disease. Molecular and therapeutic approaches.. Curr. Pharm. Des..

[16] Valero T. (2014). Mitochondrial biogenesis: pharmacological approaches.. Curr. Pharm. Des..

[17] Simmons E.C., Scholpa N.E., Schnellmann R.G. (2020). Mitochondrial biogenesis as a therapeutic target for traumatic and neurodegenerative CNS diseases.. Exp. Neurol..

[18] Atanasov N., Klisurova V., Katsarova S., Vlaĭkova E. (1988). Daily individual fluctuations of 16 clinico-chemical indices of the blood serum in hospitalized patients.. Lab. Delo.

[19] Cameron R.B., Beeson C.C., Schnellmann R.G. (2016). Development of therapeutics that induce mitochondrial biogenesis for the treatment of acute and chronic degenerative diseases.. J. Med. Chem..

[20] Dong W., Gao D., Zhang X. (2007). Mitochondria biogenesis induced by resveratrol against brain ischemic stroke.. Med. Hypotheses.

[21] Griñán-Ferré C., Bellver-Sanchis A., Izquierdo V., Corpas R., Roig-Soriano J., Chillón M., Andres-Lacueva C., Somogyvári M., Sőti C., Sanfeliu C., Pallàs M. (2021). The pleiotropic neuroprotective effects of resveratrol in cognitive decline and Alzheimer’s disease pathology: From antioxidant to epigenetic therapy.. Ageing Res. Rev..

[22] Donato A., Kagias K., Zhang Y., Hilliard M.A. (2019). Neuronal sub‐compartmentalization: a strategy to optimize neuronal function.. Biol. Rev. Camb. Philos. Soc..

[23] Cardanho-Ramos C., Morais V.A. (2021). Mitochondrial Biogenesis in Neurons: How and Where.. Int. J. Mol. Sci..

[24] Baker M.J., Frazier A.E., Gulbis J.M., Ryan M.T. (2007). Mitochondrial protein-import machinery: correlating structure with function.. Trends Cell Biol..

[25] Boguszewska K., Szewczuk M., Kaźmierczak-Barańska J., Karwowski B.T. (2020). The Similarities between human mitochondria and bacteria in the context of structure, genome, and base excision repair system.. Molecules.

[26] Fontana G.A., Gahlon H.L. (2020). Mechanisms of replication and repair in mitochondrial DNA deletion formation.. Nucleic Acids Res..

[27] Popov L.D. (2020). Mitochondrial biogenesis: An update.. J. Cell. Mol. Med..

[28] Rossi A., Pizzo P., Filadi R. (2019). Calcium, mitochondria and cell metabolism: A functional triangle in bioenergetics.. Biochim. Biophys. Acta Mol. Cell Res..

[29] Cheng C.F., Ku H.C., Lin H. (2018). PGC-1α as a pivotal factor in lipid and metabolic regulation.. Int. J. Mol. Sci..

[30] Yang Z.F., Drumea K., Mott S., Wang J., Rosmarin A.G. (2014). GABP transcription factor (nuclear respiratory factor 2) is required for mitochondrial biogenesis.. Mol. Cell. Biol..

[31] Satoh J., Kawana N., Yamamoto Y. (2013). Pathway analysis of ChIP-seq-based NRF1 target genes suggests a logical hypothesis of their involvement in the pathogenesis of neurodegenerative diseases.. Gene Regul. Syst. Bio..

[32] Biswas M., Chan J.Y. (2010). Role of Nrf1 in antioxidant response element-mediated gene expression and beyond.. Toxicol. Appl. Pharmacol..

[33] Nanjaiah H., Vallikannan B. (2019). Lutein upregulates the PGC‐1α, NRF1, and TFAM expression by AMPK activation and downregulates ROS to maintain mtDNA integrity and mitochondrial biogenesis in hyperglycemic ARPE‐19 cells and rat retina.. Biotechnol. Appl. Biochem..

[34] Graziewicz M.A., Longley M.J., Copeland W.C. (2006). DNA polymerase γ in mitochondrial DNA replication and repair.. Chem. Rev..

[35] Oláhová M., Peter B., Szilagyi Z., Diaz-Maldonado H., Singh M., Sommerville E.W., Blakely E.L., Collier J.J., Hoberg E., Stránecký V., Hartmannová H., Bleyer A.J., McBride K.L., Bowden S.A., Korandová Z., Pecinová A., Ropers H.H., Kahrizi K., Najmabadi H., Tarnopolsky M.A., Brady L.I., Weaver K.N., Prada C.E., Õunap K., Wojcik M.H., Pajusalu S., Syeda S.B., Pais L., Estrella E.A., Bruels C.C., Kunkel L.M., Kang P.B., Bonnen P.E., Mráček T., Kmoch S., Gorman G.S., Falkenberg M., Gustafsson C.M., Taylor R.W. (2021). POLRMT mutations impair mitochondrial transcription causing neurological disease.. Nat. Commun..

[36] Liu Y., Chen Z., Wang Z-H. (2021). The PPR domain of mitochondrial RNA polymerase is a ribonuclease required for mtDNA replication.. Nat. Cell Biol..

[37] Rebelo A.P., Dillon L.M., Moraes C.T. (2011). Mitochondrial DNA transcription regulation and nucleoid organization.. J. Inherit. Metab. Dis..

[38] Metodiev M.D., Lesko N., Park C.B., Cámara Y., Shi Y., Wibom R., Hultenby K., Gustafsson C.M., Larsson N.G. (2009). Methylation of 12S rRNA is necessary for in vivo stability of the small subunit of the mammalian mitochondrial ribosome.. Cell Metab..

[39] Kummer E., Ban N. (2021). Mechanisms and regulation of protein synthesis in mitochondria.. Nat. Rev. Mol. Cell Biol..

[40] Gordon D.M., Santos J.H. (2010). The emerging role of telomerase reverse transcriptase in mitochondrial DNA metabolism.. J. Nucleic Acids.

[41] Singhapol C., Pal D., Czapiewski R., Porika M., Nelson G., Saretzki G.C. (2013). Mitochondrial telomerase protects cancer cells from nuclear DNA damage and apoptosis.. PLoS One.

[42] Green P., Sharma N., Santos J. (2019). Santos JHJIjoms. Telomerase impinges on the cellular response to oxidative stress through mitochondrial ROS-mediated regulation of autophagy.. Int. J. Mol. Sci..

[43] Schmidt O., Pfanner N., Meisinger C. (2010). Mitochondrial protein import: from proteomics to functional mechanisms.. Nat. Rev. Mol. Cell Biol..

[44] Callegari S., Cruz-Zaragoza L.D., Rehling P. (2020). From TOM to the TIM23 complex – handing over of a precursor.. Biol. Chem..

[45] Zorova L.D., Popkov V.A., Plotnikov E.J. (2018). Functional significance of the mitochondrial membrane potential. Biochemistry (Moscow).. Supplement Series A: Membrane and Cell Biology.

[46] Mårtensson C.U., Priesnitz C., Song J., Ellenrieder L., Doan K.N., Boos F., Floerchinger A., Zufall N., Oeljeklaus S., Warscheid B., Becker T. (2019). Mitochondrial protein translocation-associated degradation.. Nature.

[47] Mokranjac D. (2020). How to get to the other side of the mitochondrial inner membrane – the protein import motor.. Biol. Chem..

[48] Yu L., Yang S.J. (2010). AMP-activated protein kinase mediates activity-dependent regulation of peroxisome proliferator-activated receptor γ coactivator-1α and nuclear respiratory factor 1 expression in rat visual cortical neurons.. Neuroscience.

[49] Jäger S., Handschin C., St-Pierre J., Spiegelman B.M. (2007). AMP-activated protein kinase (AMPK) action in skeletal muscle via direct phosphorylation of PGC-1α.. Proc. Natl. Acad. Sci. USA.

[50] Lee H., Zandkarimi F., Zhang Y., Meena J.K., Kim J., Zhuang L., Tyagi S., Ma L., Westbrook T.F., Steinberg G.R., Nakada D., Stockwell B.R., Gan B. (2020). Energy-stress-mediated AMPK activation inhibits ferroptosis.. Nat. Cell Biol..

[51] Fernandez-Marcos P.J., Auwerx J. (2011). Regulation of PGC-1α, a nodal regulator of mitochondrial biogenesis.. Am. J. Clin. Nutr..

[52] Delghandi M.P., Johannessen M., Moens U. (2005). The cAMP signalling pathway activates CREB through PKA, p38 and MSK1 in NIH 3T3 cells.. Cell. Signal..

[53] Cantó C., Gerhart-Hines Z., Feige J.N., Lagouge M., Noriega L., Milne J.C., Elliott P.J., Puigserver P., Auwerx J. (2009). AMPK regulates energy expenditure by modulating NAD+ metabolism and SIRT1 activity.. Nature.

[54] Mattson M.P., Gleichmann M., Cheng A. (2008). Mitochondria in neuroplasticity and neurological disorders.. Neuron.

[55] Li P.A., Hou X., Hao S. (2017). Mitochondrial biogenesis in neurodegeneration.. J. Neurosci. Res..

[56] Uittenbogaard M., Chiaramello A. (2014). Mitochondrial biogenesis: a therapeutic target for neurodevelopmental disorders and neurodegenerative diseases.. Curr. Pharm. Des..

[57] Zhang Q., Wu Y., Zhang P., Sha H., Jia J., Hu Y., Zhu J. (2012). Exercise induces mitochondrial biogenesis after brain ischemia in rats.. Neuroscience.

[58] López-Lluch G., Hunt N., Jones B., Zhu M., Jamieson H., Hilmer S., Cascajo M.V., Allard J., Ingram D.K., Navas P., de Cabo R. (2006). Calorie restriction induces mitochondrial biogenesis and bioenergetic efficiency.. Proc. Natl. Acad. Sci. USA.

[59] Komen J.C., Thorburn D.R. (2014). Turn up the power - pharmacological activation of mitochondrial biogenesis in mouse models.. Br. J. Pharmacol..

[60] Singh A., Faccenda D., Campanella M. (2021). Pharmacological advances in mitochondrial therapy.. EBioMedicine.

[61] Chodari L., Dilsiz Aytemir M., Vahedi P. (2021). Targeting mitochondrial biogenesis with polyphenol compounds.. Oxid. Med. Cell. Longev..

[62] Davinelli S., Sapere N., Visentin M., Zella D., Scapagnini G. (2013). Enhancement of mitochondrial biogenesis with polyphenols: combined effects of resveratrol and equol in human endothelial cells.. Immun. Ageing.

[63] Park S.J., Ahmad F., Philp A., Baar K., Williams T., Luo H., Ke H., Rehmann H., Taussig R., Brown A.L., Kim M.K., Beaven M.A., Burgin A.B., Manganiello V., Chung J.H. (2012). Resveratrol ameliorates aging-related metabolic phenotypes by inhibiting cAMP phosphodiesterases.. Cell.

[64] D’Errico I., Salvatore L., Murzilli S., Lo Sasso G., Latorre D., Martelli N., Egorova A.V., Polishuck R., Madeyski-Bengtson K., Lelliott C., Vidal-Puig A.J., Seibel P., Villani G., Moschetta A. (2011). Peroxisome proliferator-activated receptor-γ coactivator 1-α (PGC1α) is a metabolic regulator of intestinal epithelial cell fate.. Proc. Natl. Acad. Sci. USA.

[65] Boström P., Wu J., Jedrychowski M.P., Korde A., Ye L., Lo J.C., Rasbach K.A., Boström E.A., Choi J.H., Long J.Z., Kajimura S., Zingaretti M.C., Vind B.F., Tu H., Cinti S., Højlund K., Gygi S.P., Spiegelman B.M.A. (2012). PGC1-α-dependent myokine that drives brown-fat-like development of white fat and thermogenesis.. Nature.

[66] Handschin C., Spiegelman B.M. (2008). The role of exercise and PGC1α in inflammation and chronic disease.. Nature.

[67] Wenz T., Rossi S.G., Rotundo R.L., Spiegelman B.M., Moraes C.T. (2009). Increased muscle PGC-1α expression protects from sarcopenia and metabolic disease during aging.. Proc. Natl. Acad. Sci. USA.

[68] Piccinin E., Sardanelli A.M., Seibel P., Moschetta A., Cocco T., Villani G. (2021). PGC-1s in the Spotlight with Parkinson’s Disease.. Int. J. Mol. Sci..

[69] Mota B.C., Almpani E.V., Nikolaou M.N., Garcia-Segura M.E., Huang Y-H., Keniyopoullos R., Mazarakis N.D., Sastre M. (2020). Investigation of the effect of PGC1A gene therapy at advanced stages of Alzheimer’s disease in an animal model of amyloid pathology.. Alzheimers Dement..

[70] Yang A.J.T., Bagit A., MacPherson R.E.K. (2021). Resveratrol, metabolic dysregulation, and Alzheimer’s disease: Considerations for neurogenerative disease.. Int. J. Mol. Sci..

[71] Price N.L., Gomes A.P., Ling A.J.Y., Duarte F.V., Martin-Montalvo A., North B.J., Agarwal B., Ye L., Ramadori G., Teodoro J.S., Hubbard B.P., Varela A.T., Davis J.G., Varamini B., Hafner A., Moaddel R., Rolo A.P., Coppari R., Palmeira C.M., de Cabo R., Baur J.A., Sinclair D.A. (2012). SIRT1 is required for AMPK activation and the beneficial effects of resveratrol on mitochondrial function.. Cell Metab..

[72] Wang X.L., Li T., Li J.H., Miao S.Y., Xiao X.Z. (2017). The effects of resveratrol on inflammation and oxidative stress in a rat model of chronic obstructive pulmonary disease.. Molecules.

[73] Nishigaki A., Kido T., Kida N., Kakita-Kobayashi M., Tsubokura H., Hisamatsu Y., Okada H. (2020). Resveratrol protects mitochondrial quantity by activating SIRT1/PGC‐1α expression during ovarian hypoxia.. Reprod. Med. Biol..

[74] Zhang T., Chi Y., Ren Y., Du C., Shi Y., Li Y. (2019). Resveratrol reduces oxidative stress and apoptosis in podocytes via Sir2-related enzymes, sirtuins1 (SIRT1)/peroxisome proliferator-activated receptor γ co-activator 1α (PGC-1α).. Axis. Med. Sci. Monit..

[75] Fang W., Wang C., He Y., Zhou Y., Peng X., Liu S. (2018). Resveratrol alleviates diabetic cardiomyopathy in rats by improving mitochondrial function through PGC-1α deacetylation.. Acta Pharmacol. Sin..

[76] Lagouge M., Argmann C., Gerhart-Hines Z., Meziane H., Lerin C., Daussin F., Messadeq N., Milne J., Lambert P., Elliott P., Geny B., Laakso M., Puigserver P., Auwerx J. (2006). Resveratrol improves mitochondrial function and protects against metabolic disease by activating SIRT1 and PGC-1alpha.. Cell.

[77] Ljubicic V., Burt M., Lunde J.A., Jasmin B.J. (2014). Resveratrol induces expression of the slow, oxidative phenotype in mdx mouse muscle together with enhanced activity of the SIRT1-PGC-1α axis.. Am. J. Physiol. Cell Physiol..

[78] Fu B., Zhao J., Peng W., Wu H., Zhang Y. (2017). Resveratrol rescues cadmium-induced mitochondrial injury by enhancing transcriptional regulation of PGC-1α and SOD2 via the Sirt3/FoxO3a pathway in TCMK-1 cells.. Biochem. Biophys. Res. Commun..

[79] Zhou J., Yang Z., Shen R., Zhong W., Zheng H., Chen Z., Tang J., Zhu J. (2021). Resveratrol improves mitochondrial biogenesis function and activates PGC-1α pathway in a preclinical model of early brain injury following subarachnoid hemorrhage.. Front. Mol. Biosci..

[80] Baldelli S., Aquilano K., Ciriolo M. (2014). PGC-1α buffers ROS-mediated removal of mitochondria during myogenesis.. Cell Death Dis..

[81] St-Pierre J., Drori S., Uldry M., Silvaggi J.M., Rhee J., Jäger S., Handschin C., Zheng K., Lin J., Yang W., Simon D.K., Bachoo R., Spiegelman B.M. (2006). Suppression of reactive oxygen species and neurodegeneration by the PGC-1 transcriptional coactivators.. Cell.

[82] Chuang Y.C., Chen S.D., Hsu C.Y., Chen S.F., Chen N.C., Jou S.B. (2019). Resveratrol promotes mitochondrial biogenesis and protects against seizure-induced neuronal cell damage in the hippocampus following status epilepticus by activation of the PGC-1α signaling pathway.. Int. J. Mol. Sci..

[83] Chen S., Fan Q., Li A., Liao D., Ge J., Laties A.M., Zhang X. (2013). Dynamic mobilization of PGC-1α mediates mitochondrial biogenesis for the protection of RGC-5 cells by resveratrol during serum deprivation.. Apoptosis.

[84] Harry G.J. (2021). Microglia in neurodegenerative events—an initiator or a significant other?. Int. J. Mol. Sci..

[85] Yang X., Xu S., Qian Y., Xiao Q. (2017). Resveratrol regulates microglia M1/M2 polarization via PGC-1α in conditions of neuroinflammatory injury.. Brain Behav. Immun..

[86] Gureev A.P., Shaforostova E.A., Popov V.N. (2019). Regulation of mitochondrial biogenesis as a way for active longevity: Interaction between the Nrf2 and PGC-1α signaling pathways.. Front. Genet..

[87] Evans M.J., Scarpulla R.C. (1990). NRF-1: a trans-activator of nuclear-encoded respiratory genes in animal cells.. Genes Dev..

[88] Yan X., Shen Z., Yu D., Zhao C., Zou H., Ma B., Dong W., Chen W., Huang D., Yu Z. (2022). Nrf2 contributes to the benefits of exercise interventions on age-related skeletal muscle disorder via regulating Drp1 stability and mitochondrial fission.. Free Radic. Biol. Med..

[89] Scarpulla R.C. (2008). Transcriptional paradigms in mammalian mitochondrial biogenesis and function.. Physiol. Rev..

[90] Saha S., Buttari B., Panieri E., Profumo E., Saso L. (2020). An overview of Nrf2 signaling pathway and its role in inflammation.. Molecules.

[91] Johri A., Chandra A., Flint Beal M. (2013). PGC-1α, mitochondrial dysfunction, and Huntington’s disease.. Free Radic. Biol. Med..

[92] Ramsey C.P., Glass C.A., Montgomery M.B., Lindl K.A., Ritson G.P., Chia L.A., Hamilton R.L., Chu C.T., Jordan-Sciutto K.L. (2007). Expression of Nrf2 in neurodegenerative diseases.. J. Neuropathol. Exp. Neurol..

[93] Ikram M., Park T.J., Ali T., Kim M.O. (2020). Antioxidant and neuroprotective effects of caffeine against Alzheimer’s and Parkinson’s disease: insight into the role of Nrf-2 and A2AR signaling.. Antioxidants.

[94] Uruno A., Matsumaru D., Ryoke R., Saito R., Kadoguchi S., Saigusa D., Saito T., Saido T.C., Kawashima R., Yamamoto M. (2020). Nrf2 suppresses oxidative stress and inflammation in App knock-in Alzheimer’s disease model mice.. Mol. Cell. Biol..

[95] Sheng B., Wang X., Su B., Lee H., Casadesus G., Perry G., Zhu X. (2012). Impaired mitochondrial biogenesis contributes to mitochondrial dysfunction in Alzheimer’s disease.. J. Neurochem..

[96] Franco-Iborra S., Vila M., Perier C. (2018). Mitochondrial quality control in neurodegenerative diseases: focus on Parkinson’s disease and Huntington’s disease.. Front. Neurosci..

[97] Taherzadeh-Fard E., Saft C., Akkad D.A., Wieczorek S., Haghikia A., Chan A., Epplen J.T., Arning L. (2011). PGC-1alpha downstream transcription factors NRF-1 and TFAM are genetic modifiers of Huntington disease.. Mol. Neurodegener..

[98] Yang J., Huang J., Shen C., Cheng W., Yu P., Wang L., Tang F., Guo S., Yang Q., Zhang J. (2018). Resveratrol treatment in different time-attenuated neuronal apoptosis after oxygen and glucose deprivation/reoxygenation via enhancing the activation of Nrf-2 signaling pathway in vitro.. Cell Transplant..

[99] Abdel-Aleem G.A., Khaleel E.F., Mostafa D.G., Elberier L.K. (2016). Neuroprotective effect of resveratrol against brain ischemia reperfusion injury in rats entails reduction of DJ-1 protein expression and activation of PI3K/Akt/GSK3b survival pathway.. Arch. Physiol. Biochem..

[100] Kesherwani V., Atif F., Yousuf S., Agrawal S.K. (2013). Resveratrol protects spinal cord dorsal column from hypoxic injury by activating Nrf-2.. Neuroscience.

[101] Zhao Q., Tian Z., Zhou G., Niu Q., Chen J., Li P., Dong L., Xia T., Zhang S., Wang A. (2020). SIRT1-dependent mitochondrial biogenesis supports therapeutic effects of resveratrol against neurodevelopment damage by fluoride.. Theranostics.

[102] Ho D.J., Calingasan N.Y., Wille E., Dumont M., Beal M.F. (2010). Resveratrol protects against peripheral deficits in a mouse model of Huntington’s disease.. Exp. Neurol..

[103] Mattingly K.A., Klinge C.M. (2012). Diesel exhaust particulate extracts inhibit transcription of nuclear respiratory factor-1 and cell viability in human umbilical vein endothelial cells.. Arch. Toxicol..

[104] Nirwane A., Majumdar A. (2016). Resveratrol and pterostilbene attenuated smokeless tobacco induced cardiovascular aberrations in estrogen deficient female rats.. Toxicol. Res. (Camb.).

[105] Chang H.C., Guarente L. (2014). SIRT1 and other sirtuins in metabolism.. Trends Endocrinol. Metab..

[106] Rodgers J.T., Lerin C., Haas W., Gygi S.P., Spiegelman B.M., Puigserver P. (2005). Nutrient control of glucose homeostasis through a complex of PGC-1α and SIRT1.. Nature.

[107] Liu Y., Dentin R., Chen D., Hedrick S., Ravnskjaer K., Schenk S., Milne J., Meyers D.J., Cole P., Iii J.Y., Olefsky J., Guarente L., Montminy M. (2008). A fasting inducible switch modulates gluconeogenesis via activator/coactivator exchange.. Nature.

[108] Purushotham A., Schug T.T., Xu Q., Surapureddi S., Guo X., Li X. (2009). Hepatocyte-specific deletion of SIRT1 alters fatty acid metabolism and results in hepatic steatosis and inflammation.. Cell Metab..

[109] Donmez G., Outeiro T.F. (2013). SIRT1 and SIRT2: emerging targets in neurodegeneration.. EMBO Mol. Med..

[110] Manjula R., Anuja K., Alcain F.J. (2021). SIRT1 and SIRT2 activity control in neurodegenerative diseases.. Front. Pharmacol..

[111] Tanno M., Sakamoto J., Miura T., Shimamoto K., Horio Y. (2007). Nucleocytoplasmic shuttling of the NAD+-dependent histone deacetylase SIRT1.. J. Biol. Chem..

[112] Donmez G. (2012). The Effects of SIRT1 on Alzheimer’s Disease Models.. Int. J. Alzheimers Dis..

[113] Li X., Feng Y., Wang X.X., Truong D., Wu Y.C. (2020). The critical role of SIRT1 in Parkinson’s disease: mechanism and therapeutic considerations.. Aging Dis..

[114] Jeong H., Cohen D.E., Cui L., Supinski A., Savas J.N., Mazzulli J.R., Yates J.R., Bordone L., Guarente L., Krainc D. (2012). Sirt1 mediates neuroprotection from mutant huntingtin by activation of the TORC1 and CREB transcriptional pathway.. Nat. Med..

[115] Duan W. (2013). Targeting sirtuin-1 in Huntington’s disease: rationale and current status.. CNS Drugs.

[116] Bagul P., Katare P., Bugga P., Dinda A., Banerjee S.K. (2018). SIRT-3 modulation by resveratrol improves mitochondrial oxidative phosphorylation in diabetic heart through deacetylation of TFAM.. Cells.

[117] Denu J.M. (2012). Fortifying the link between SIRT1, resveratrol, and mitochondrial function.. Cell Metab..

[118] Xu Y., Nie L., Yin Y.G., Tang J.L., Zhou J.Y., Li D.D., Zhou S.W. (2012). Resveratrol protects against hyperglycemia-induced oxidative damage to mitochondria by activating SIRT1 in rat mesangial cells.. Toxicol. Appl. Pharmacol..

[119] Tanno M., Kuno A., Yano T., Miura T., Hisahara S., Ishikawa S., Shimamoto K., Horio Y. (2010). Induction of manganese superoxide dismutase by nuclear translocation and activation of SIRT1 promotes cell survival in chronic heart failure.. J. Biol. Chem..

[120] Olmos Y., Sánchez-Gómez F.J., Wild B., García-Quintans N., Cabezudo S., Lamas S., Monsalve M. (2013). SirT1 regulation of antioxidant genes is dependent on the formation of a FoxO3a/PGC-1α complex.. Antioxid. Redox Signal..

[121] Gomes B.A.Q., Silva J.P.B., Romeiro C.F.R., dos Santos S.M., Rodrigues C.A., Gonçalves P.R., Sakai J.T., Mendes P.F.S., Varela E.L.P., Monteiro M.C. (2018). Neuroprotective mechanisms of resveratrol in Alzheimer’s disease: Role of SIRT1.. Oxid. Med. Cell. Longev..

[122] Venigalla M., Sonego S., Gyengesi E., Sharman M.J., Münch G. (2016). Novel promising therapeutics against chronic neuroinflammation and neurodegeneration in Alzheimer’s disease.. Neurochem. Int..

[123] Liu Y., Chen X., Li J. (2017). Resveratrol protects against oxidized low-density lipoprotein-induced human umbilical vein endothelial cell apoptosis via inhibition of mitochondrial-derived oxidative stress.. Mol. Med. Rep..

[124] Corpas R., Griñán-Ferré C., Rodríguez-Farré E., Pallàs M., Sanfeliu C. (2019). Resveratrol induces brain resilience against Alzheimer neurodegeneration through proteostasis enhancement.. Mol. Neurobiol..

[125] Zhang L., Tu R., Wang Y., Hu Y., Li X., Cheng X., Yin Y., Li W., Huang H. (2017). Early-life exposure to lead induces cognitive impairment in elder mice targeting sirt1 phosphorylation and oxidative alterations.. Front. Physiol..

[126] Porquet D., Griñán-Ferré C., Ferrer I., Camins A., Sanfeliu C., del Valle J., Pallàs M. (2014). Neuroprotective role of trans-resveratrol in a murine model of familial Alzheimer’s disease.. J. Alzheimers Dis..

[127] Bai L., Liu R., Wang R., Xin Y., Wu Z., Ba Y., Zhang H., Cheng X., Zhou G., Huang H. (2021). Attenuation of Pb-induced Aβ generation and autophagic dysfunction via activation of SIRT1: Neuroprotective properties of resveratrol.. Ecotoxicol. Environ. Saf..

[128] Wang R., Zhang Y., Li J., Zhang C. (2017). Resveratrol ameliorates spatial learning memory impairment induced by Aβ 1–42 in rats.. Neuroscience.

[129] Abozaid O.A.R., Sallam M.W., El-Sonbaty S., Aziza S., Emad B., Ahmed E.S.A. (2022). Resveratrol-selenium nanoparticles alleviate neuroinflammation and neurotoxicity in a rat model of Alzheimer’s disease by regulating Sirt1/miRNA-134/GSK3β expression.. Biol. Trace Elem. Res..

[130] Simão F., Matté A., Pagnussat A.S., Netto C.A., Salbego C.G. (2012). Resveratrol preconditioning modulates inflammatory response in the rat hippocampus following global cerebral ischemia.. Neurochem. Int..

[131] Moussa C., Hebron M., Huang X., Ahn J., Rissman R.A., Aisen P.S., Turner R.S. (2017). Resveratrol regulates neuro-inflammation and induces adaptive immunity in Alzheimer’s disease.. J. Neuroinflammation.

[132] Anton S.D., Ebner N., Dzierzewski J.M., Zlatar Z.Z., Gurka M.J., Dotson V.M., Kirton J., Mankowski R.T., Marsiske M., Manini T.M. (2018). Effects of 90 days of resveratrol supplementation on cognitive function in elders: A pilot study.. J. Altern. Complement. Med..

[133] Wang X., Ma S., Yang B., Huang T., Meng N., Xu L., Xing Q., Zhang Y., Zhang K., Li Q., Zhang T., Wu J., Yang G.L., Guan F., Wang J. (2018). Resveratrol promotes hUC-MSCs engraftment and neural repair in a mouse model of Alzheimer’s disease.. Behav. Brain Res..

[134] Gómez-Benito M., Granado N., García-Sanz P., Michel A., Dumoulin M., Moratalla R. (2020). Modeling Parkinson’s disease with the alpha-synuclein protein.. Front. Pharmacol..

[135] Jin F., Wu Q., Lu Y.F., Gong Q.H., Shi J.S. (2008). Neuroprotective effect of resveratrol on 6-OHDA-induced Parkinson’s disease in rats.. Eur. J. Pharmacol..

[136] Adedara A.O., Babalola A.D., Stephano F., Awogbindin I.O., Olopade J.O., Rocha J.B.T., Whitworth A.J., Abolaji A.O. (2022). An assessment of the rescue action of resveratrol in parkin loss of function-induced oxidative stress in Drosophila melanogaster.. Sci. Rep..

[137] Naia L., Rosenstock T.R., Oliveira A.M., Oliveira-Sousa S.I., Caldeira G.L., Carmo C., Laço M.N., Hayden M.R., Oliveira C.R., Rego A.C. (2017). Comparative mitochondrial-based protective effects of resveratrol and nicotinamide in Huntington’s disease models.. Mol. Neurobiol..

[138] Herzig S., Shaw R.J. (2018). AMPK: guardian of metabolism and mitochondrial homeostasis.. Nat. Rev. Mol. Cell Biol..

[139] Zong H., Ren J.M., Young L.H., Pypaert M., Mu J., Birnbaum M.J., Shulman G.I. (2002). AMP kinase is required for mitochondrial biogenesis in skeletal muscle in response to chronic energy deprivation.. Proc. Natl. Acad. Sci. USA.

[140] Marinangeli C., Didier S., Vingtdeux V. (2016). AMPK in neurodegenerative diseases: implications and therapeutic perspectives.. Curr. Drug Targets.

[141] Muraleedharan R., Dasgupta B. (2022). AMPK in the brain: Its roles in glucose and neural metabolism.. FEBS J..

[142] Hwang J.T., Kwon D.Y., Park O.J., Kim M.S. (2008). Resveratrol protects ROS-induced cell death by activating AMPK in H9c2 cardiac muscle cells.. Genes Nutr..

[143] Patel M.I., Gupta A., Dey C.S. (2011). Potentiation of neuronal insulin signaling and glucose uptake by resveratrol: the involvement of AMPK.. Pharmacol. Rep..

[144] Vlavcheski F., Den Hartogh D.J., Giacca A., Tsiani E. (2020). Amelioration of high-insulin-induced skeletal muscle cell insulin resistance by resveratrol is linked to activation of AMPK and restoration of GLUT4 translocation.. Nutrients.

[145] Kim M.Y., Lim J.H., Youn H.H., Hong Y.A., Yang K.S., Park H.S., Chung S., Koh S.H., Shin S.J., Choi B.S., Kim H.W., Kim Y.S., Lee J.H., Chang Y.S., Park C.W. (2013). Resveratrol prevents renal lipotoxicity and inhibits mesangial cell glucotoxicity in a manner dependent on the AMPK–SIRT1–PGC1α axis in db/db mice.. Diabetologia.

[146] Kang R.R., Sun Q., Chen K.G., Cao Q.T., Liu C., Liu K., Ma Z., Deng Y., Liu W., Xu B. (2020). Resveratrol prevents benzo(a)pyrene-induced disruption of mitochondrial homeostasis via the AMPK signaling pathway in primary cultured neurons.. Environ. Pollut..

[147] Lin C.H., Nicol C.J.B., Cheng Y.C., Yen C., Wang Y.S., Chiang M.C. (2020). Neuroprotective effects of resveratrol against oxygen glucose deprivation induced mitochondrial dysfunction by activation of AMPK in SH-SY5Y cells with 3D gelatin scaffold.. Brain Res..

[148] Dasgupta B., Milbrandt J. (2007). Resveratrol stimulates AMP kinase activity in neurons.. Proc. Natl. Acad. Sci. USA.

[149] Wu Y., Li X., Zhu J.X., Xie W., Le W., Fan Z., Jankovic J., Pan T. (2011). Resveratrol-activated AMPK/SIRT1/autophagy in cellular models of Parkinson’s disease.. Neurosignals.

[150] Ferretta A., Gaballo A., Tanzarella P., Piccoli C., Capitanio N., Nico B., Annese T., Di Paola M., Dell’Aquila C., De Mari M., Ferranini E., Bonifati V., Pacelli C., Cocco T. (2014). Effect of resveratrol on mitochondrial function: Implications in parkin-associated familiar Parkinson’s disease.. Biochim. Biophys. Acta Mol. Basis Dis..

[151] Pineda-Ramírez N., Alquisiras-Burgos I., Ortiz-Plata A., Ruiz-Tachiquín M.E., Espinoza-Rojo M., Aguilera P. (2020). Resveratrol Activates Neuronal Autophagy Through AMPK in the Ischemic Brain.. Mol. Neurobiol..

[152] Jang B.G., Lee J., Choi B., Koh Y.H., Kim M.J. (2021). Unexpected beta-amyloid production by middle doses of resveratrol through stabilization of APP protein and AMPK-mediated inhibition of trypsin-like proteasome activity in a cell model of Alzheimer’s disease.. Food Chem. Toxicol..

[153] Porquet D., Casadesús G., Bayod S., Vicente A., Canudas A.M., Vilaplana J., Pelegrí C., Sanfeliu C., Camins A., Pallàs M., del Valle J. (2013). Dietary resveratrol prevents Alzheimer’s markers and increases life span in SAMP8.. Age (Omaha).

[154] Vingtdeux V., Giliberto L., Zhao H., Chandakkar P., Wu Q., Simon J.E., Janle E.M., Lobo J., Ferruzzi M.G., Davies P., Marambaud P. (2010). AMP-activated protein kinase signaling activation by resveratrol modulates amyloid-beta peptide metabolism.. J. Biol. Chem..

[155] Qi Y., Shang L., Liao Z., Su H., Jing H., Wu B., Bi K., Jia Y. (2019). Intracerebroventricular injection of resveratrol ameliorated Aβ-induced learning and cognitive decline in mice.. Metab. Brain Dis..

[156] Shaito A., Posadino A.M., Younes N., Hasan H., Halabi S., Alhababi D., Al-Mohannadi A., Abdel-Rahman W.M., Eid A.H., Nasrallah G.K., Pintus G. (2020). Potential adverse effects of resveratrol: A literature review.. Int. J. Mol. Sci..

[157] Posadino A.M., Giordo R., Cossu A., Nasrallah G.K., Shaito A., Abou-Saleh H., Eid A.H., Pintus G. (2019). Flavin oxidase-induced ROS generation modulates PKC biphasic effect of resveratrol on endothelial cell survival.. Biomolecules.

[158] Posadino A.M., Cossu A., Giordo R., Zinellu A., Sotgia S., Vardeu A., Hoa P.T., Nguyen L.H.V., Carru C., Pintus G. (2015). Resveratrol alters human endothelial cells redox state and causes mitochondrial-dependent cell death.. Food Chem. Toxicol..

[159] Giordo R., Cossu A., Pasciu V., Hoa P.T., Posadino A.M., Pintus G. (2013). Different redox response elicited by naturally occurring antioxidants in human endothelial cells.. Open Biochem. J..

[160] Pasciu V., Posadino A.M., Cossu A., Sanna B., Tadolini B., Gaspa L., Marchisio A., Dessole S., Capobianco G., Pintus G. (2010). Akt downregulation by flavin oxidase-induced ROS generation mediates dose-dependent endothelial cell damage elicited by natural antioxidants.. Toxicol. Sci..

[161] Klinge C.M. (2020). Estrogenic control of mitochondrial function.. Redox Biol..

[162] Cho Y., Hazen B.C., Russell A.P., Kralli A. (2013). Peroxisome proliferator-activated receptor γ coactivator 1 (PGC-1)- and estrogen-related receptor (ERR)-induced regulator in muscle 1 (Perm1) is a tissue-specific regulator of oxidative capacity in skeletal muscle cells.. J. Biol. Chem..

[163] Tang Y., Min Z., Xiang X.J., Liu L., Ma Y.L., Zhu B.L., Song L., Tang J., Deng X.J., Yan Z., Chen G.J. (2018). Estrogen-related receptor alpha is involved in Alzheimer’s disease-like pathology.. Exp. Neurol..

[164] Ren Y., Jiang H., Ma D., Nakaso K., Feng J. (2011). Parkin degrades estrogen-related receptors to limit the expression of monoamine oxidases.. Hum. Mol. Genet..

[165] Chaturvedi R.K., Calingasan N.Y., Yang L., Hennessey T., Johri A., Beal M.F. (2010). Impairment of PGC-1alpha expression, neuropathology and hepatic steatosis in a transgenic mouse model of Huntington’s disease following chronic energy deprivation.. Hum. Mol. Genet..

[166] Naia L., Rego A.C. (2015). Sirtuins: double players in Huntington’s disease.. Biochim. Biophys. Acta Mol. Basis Dis..

[167] Lopes Costa A., Le Bachelier C., Mathieu L., Rotig A., Boneh A., De Lonlay P., Tarnopolsky M.A., Thorburn D.R., Bastin J., Djouadi F. (2014). Beneficial effects of resveratrol on respiratory chain defects in patients’ fibroblasts involve estrogen receptor and estrogen-related receptor alpha signaling.. Hum. Mol. Genet..

[168] Lu Y., Lu X., Wang L., Yang W. (2019). Resveratrol attenuates high fat diet-induced mouse cardiomyopathy through upregulation of estrogen related receptor-α.. Eur. J. Pharmacol..

[169] Dratwa M., Wysoczańska B., Łacina P., Kubik T., Bogunia-Kubik K. (2020). TERT—Regulation and roles in cancer formation.. Front. Immunol..

[170] Saretzki G. (2014). Extra-telomeric functions of human telomerase: cancer, mitochondria and oxidative stress.. Curr. Pharm. Des..

[171] Lionaki E., Gkikas I., Tavernarakis N. (2016). Differential protein distribution between the nucleus and mitochondria: implications in aging.. Front. Genet..

[172] Spilsbury A., Miwa S., Attems J., Saretzki G. (2015). The role of telomerase protein TERT in Alzheimer’s disease and in tau-related pathology in vitro.. J. Neurosci..

[173] Saretzki G., Wan T. (2021). Telomerase in brain: The new kid on the block and its role in neurodegenerative diseases.. Biomedicines.

[174] Huang P., Riordan S.M., Heruth D.P., Grigoryev D.N., Zhang L.Q., Ye S.Q. (2015). A critical role of nicotinamide phosphoribosyltransferase in human telomerase reverse transcriptase induction by resveratrol in aortic smooth muscle cells.. Oncotarget.

[175] Wang X-B., Zhu L., Huang J., Yin Y.G., Kong X.Q., Rong Q.F., Shi A.W., Cao K.J. (2011). Resveratrol-induced augmentation of telomerase activity delays senescence of endothelial progenitor cells.. Chin. Med. J. (Engl.).

[176] Martí-Centelles R., Falomir E., Murga J., Carda M., Marco J.A. (2015). Inhibitory effect of cytotoxic stilbenes related to resveratrol on the expression of the VEGF, hTERT and c-Myc genes.. Eur. J. Med. Chem..

[177] Kim S.H., Cho K.H., Kim Y.N., Jeong B.Y., Park C.G., Hur G.M., Lee H.Y. (2016). Resveratrol attenuates norepinephrine-induced ovarian cancer invasiveness through downregulating hTERT expression.. Arch. Pharm. Res..

[178] Sheikhha M.H., Mirzazadeh A., Kheirollahi M., Farashahi E., Sadeghian-Nodoushan F., Aflatoonian B. (2017). Assessment effects of resveratrol on human telomerase reverse transcriptase messenger ribonucleic acid transcript in human glioblastoma.. Adv. Biomed. Res..

[179] Sun Q., Kang R.R., Chen K.G., Liu K., Ma Z., Liu C., Deng Y., Liu W., Xu B. (2021). Sirtuin 3 is required for the protective effect of Resveratrol on Manganese‐induced disruption of mitochondrial biogenesis in primary cultured neurons.. J. Neurochem..

[180] Tian Z.Y., Chen J.W., Zhou G.Y., Li P., Zhou Q., Luo C., Zhang S., Wang A.G. (2018). The effects of resveratrol on mitochondrial biogenesis dysfunction induced by fluoride in human neuroblastoma SH-SY5Y cells.. Zhonghua Lao Dong Wei Sheng Zhi Ye Bing Za Zhi.

[181] Salehi B., Mishra A., Nigam M., Sener B., Kilic M., Sharifi-Rad M., Fokou P., Martins N., Sharifi-Rad J. (2018). Resveratrol: A double-edged sword in health benefits.. Biomedicines.

[182] Giordo R., Wehbe Z., Posadino A.M., Erre G.L., Eid A.H., Mangoni A.A., Pintus G. (2022). Disease-associated regulation of non-coding RNAs by resveratrol: Molecular insights and therapeutic applications.. Front. Cell Dev. Biol..

